# Dynamic Evolution of Mass and Physical Properties of Atmospheric Organic Aerosol Under Solar Irradiance

**DOI:** 10.1021/acs.est.5c16671

**Published:** 2026-02-18

**Authors:** Bin Bai, Gregory W. Vandergrift, Yutong Liang, Yaowei Li, Zezhen Cheng, Yuchen Wang, Nara Shin, Frank Keutsch, Andrew Lambe, Swarup China, Nga L. Ng, Pengfei Liu

**Affiliations:** † School of Earth and Atmospheric Sciences, 1372 Georgia Institute of Technology, Atlanta, Georgia 30332, United States; ‡ Environmental Molecular Sciences Laboratory, 6865 Pacific Northwest National Laboratory, Richland, Washington 99354, United States; § School of Chemical and Biomolecular Engineering, Georgia Institute of Technology, Atlanta, Georgia 30332, United States; ∥ Thrust of Sustainable Energy and Environment, The Hong Kong University of Science and Technology (Guangzhou), Guangzhou, Guangdong 511453, China; ⊥ School of Engineering and Applied Sciences, 124077 Harvard University, Cambridge, Massachusetts 02138, United States; # College of Environmental Science and Engineering, Hunan University, Changsha, Hunan 410082, China; ○ 53777 Aerodyne Research, Billerica, Massachusetts 01821, United States; ⬢ School of Engineering and Applied Sciences and Department of Chemistry and Chemical Biology and Department of Earth and Planetary Sciences, Harvard University, Cambridge, Massachusetts 02138, United States; ⬡ School of Earth and Atmospheric Sciences and School of Chemical and Biomolecular Engineering and School of Civil and Environmental Engineering, Georgia Institute of Technology, Atlanta, Georgia 30332, United States

**Keywords:** photolytic aging, volatility distribution, hygroscopicity, quartz crystal microbalance (QCM), nano-DESI HRMS, elemental ratios, low-volatility products

## Abstract

Organic aerosol (OA) particles constitute a substantial fraction of submicron particulate mass in the atmosphere and play a critical role in climate system. OA undergoes dynamic aging processes in the atmosphere, with photolytic aging induced by ultraviolet solar irradiance being an important yet poorly characterized mechanism. Knowledge gaps persist regarding the role of volatility transformations during photolytic aging on the OA mass decay kinetics and the evolution of climate-relevant properties, such as hygroscopicity, hindering the model evaluation of OA spatiotemporal distributions and atmospheric budgets. In this study, we conduct isothermal photolytic aging experiments on both laboratory-generated secondary organic aerosols and ambient-collected particles from urban Atlanta, utilizing a high-sensitivity Quartz Crystal Microbalance. Our results reveal that photolytic aging reduces 40–66% of the low-volatility OA mass with lifetimes ranging from 8 to 200 h under solar irradiance, and 44–92% of the photolytic mass loss is through slow evaporation of semi- or intermediate-volatile products, kinetically limited by their volatility. We observe up to ±50% changes in OA hygroscopicity with the transformation of fresh OA to photorecalcitrant low-volatility products, associated with changes in oxygen-to-carbon ratio and molecular weight. A kinetic model incorporating photolytic volatility transformation provides a cohesive explanation for the observed photolysis-induced changes in mass, volatility, and hygroscopicity. Our results can help constrain model representation of the dynamic evolutions of mass and climate-relevant properties during photolytic aging processes of the ambient OA, improving our understanding of OA atmospheric behavior and climate impact.

## Graphical abstract

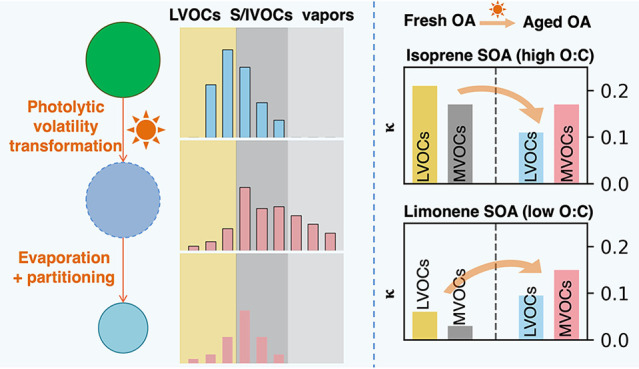

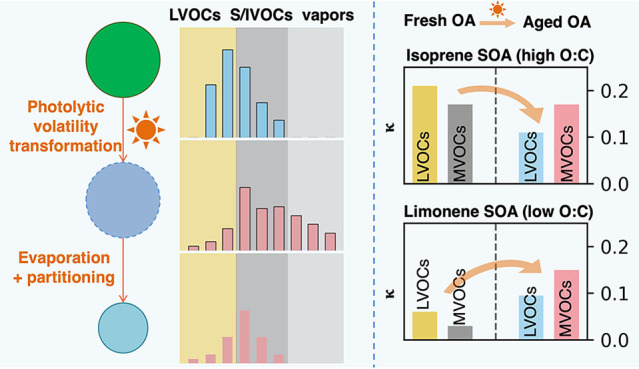

## 1 Introduction

Organic aerosol (OA) material constitutes 20–90% of the submicron particulate mass in the terrestrial atmosphere.^1^ A major fraction of OA is produced secondarily by the atmospheric oxidation of volatile organic compounds (VOCs), dominantly emitted from biogenic sources.^2^ OA can affect climate directly by scattering and absorbing solar radiation and indirectly by serving as cloud condensation nuclei (CCN), thereby altering cloud albedo and lifetime.^2,3^

The atmospheric lifetime of OA against wet and dry deposition spans from several days in the planetary boundary layer to weeks in the free troposphere.^4^ During this period, OA mass and properties undergo dynamical evolution through various aging processes, such as gas–particle repartitioning,^5^ heterogeneous oxidation,^6–9^ oligomerization,^10–12^ cloudwater processing,^13–15^ and in-particle photochemical reactions driven by ultraviolet (UV) light.^16–24^ OA composition such as carbonyls and peroxides can absorb UV light,^25,26^ which is energetic enough to initiate complex aging reactions such as direct fragmentations^27–29^ and photosensitization initiated secondary reactions. For example, triplet excited states of organic compounds from the irradiation of light-absorbing organics can oxidize organic molecules and generate other radicals upon reactions with O_2_.^30–32^ This UV light-induced aging process, referred to as *OA photolytic aging* herein, might substantially reduce the lifetime and mass loading of atmospheric OA. Global modeling indicated that photolytic loss plays a crucial role in compensating for the overestimation of the atmospheric OA budget and in altering the vertical and spatial distribution of OA.^4,33,34^

Laboratory experiments have directly quantified particle mass changes induced by UV light,^16,19–21,23,25,35–38^ suggesting significant particle mass loss, although a fraction of OA mass can resist UV degradation (“photo-recalcitrant”). Several studies have identified various low-carbon-number vapors evaporated from OA during photolytic aging.^17,19,22,39^ Properties such as light absorption and viscosity of OA were found to change after photolytic aging.^21,40^ However, information regarding the intermediate stages and time-dependent evolution of OA properties during photolytic aging is still limited, restricting the application of laboratory findings in model simulation to evaluate the climate effect of OA. For example, the evaporation or partitioning of semi- or intermediate-volatile organic compounds (S/IVOCs) play a critical role in interpreting photolysis-induced mass decay kinetics and comprehending chemical transformations.

Hygroscopicity, as an important climate-relevant physical property, determines how aerosol particles interact with water vapor, and further affects aerosol water content, visibility, and the ability of aerosol particles to serve as CCN.^41^ Previous research has shown that the CCN activity of α-pinene and naphthalene secondary organic aerosols (SOA) extracts in bulk aqueous phase increases rapidly upon exposure to UVB irradiation.^42^ However, how photolytic aging influences OA particle hygroscopicity under atmospherically relevant humidity conditions remains unknown. Moreover, previous photolytic aging studies focused on laboratory generated OA, leaving the kinetics and property changes of ambient OA under photolytic aging insufficiently explored.^22^ As a result, substantial uncertainty exists when attempting to reconcile laboratory results with OA in ambient atmosphere.

In this study, we conducted isothermal photolytic aging experiments for both laboratory-generated SOA from biogenic precursors (isoprene and limonene) and ambient-sampled particles in urban Atlanta, United States, using a high-sensitivity quartz-crystal microbalance (QCM), combined with chemical analysis using nanospray desorption electrospray ionization high resolution mass spectrometry (Nano-DESI HRMS) directly on QCM sensors. We developed a new experimental procedure within the QCM to simultaneously measure the changes in mass, volatility, and hygroscopicity of OA samples during photolytic aging. We built a new thermodynamic volatility model that incorporates photolytic volatility transformation, and successfully modeled the measured mass decay, volatility, and hygroscopicity profiles during photolytic aging within a unified framework.

## 2 Materials and Methods

### 2.1 SOA Generation, Collection and Measurement

SOA were generated through OH-initiated isoprene photooxidation in the absence of nitrogen oxides (NO_*x*_) and limonene ozonolysis in a Potential Aerosol Mass Oxidation Flow Reactor (PAM OFR, Aerodyne Research). Organic vapors were introduced via a syringe pump. Detailed information on PAM OFR can be found elsewhere.^43^ In brief, ozone (O_3_) was generated by passing the zero air through a mercury lamp (λ = 185 nm) externally, and OH radical was generated by irradiating the injected ozone with internal mercury lamps (λ = 254 nm). The average residence time inside PAM OFR was ∼132 s for an 8 Lpm total flow rate, which was sufficient for the homogeneous formation of SOA. Water vapor was introduced using a water bubbler. The photochemical ages of the precursor oxidation were characterized using a kinetic model.^44,45^ For specific experimental conditions regarding the generation of SOA in the PAM OFR, refer to Table S1.

To collect the SOA particles, an electrostatic precipitator system downstream of the PAM OFR was utilized. The generated SOA was charged by a Corona Charger (IONER CC-8020) and then deposited onto a quartz crystal (14 mm diameter) using an electrostatic precipitator (TSI Nanometer Aerosol Sampler 3089) with a flow rate of 1.0 L per minute (Figure S1). This process allowed the deposited SOA to grow as a uniform thin film on the substrates.^46^ This sampling method with improved sampling efficiency of 10–50% enabled us to extend this technique to ambient aerosol particles sampling with lower concentrations than laboratory conditions.

The aerosol particles were characterized by a scanning mobility particle sizer (SMPS model 3938, with wide-range differential mobility analyzer 3083, TSI Inc.) and an aerosol mass spectrometer (HR-ToF-AMS, Aerodyne Research). O/C and H/C ratios were calculated using PIKA v1.25c (Igor 8.0.3), following the methodology in Canagaratna et al.^47^ The particle density was estimated using the parametrization described in Kuwata et al.^48^ Detailed information regarding the laboratory-generated SOA can be found in Table S2.

### 2.2 Ambient Particle Collection

Ambient particles were collected using the same collection technique employed for the laboratory-generated SOA. The collection took place on the rooftop of the Ford Environmental Sciences and Technology building at Georgia Institute of Technology in Atlanta, Georgia in August 2022.^49^ Simultaneous HR-ToF-AMS measurements were conducted during sample collection. The averaged submicron particle concentration measured by HR-ToF-AMS was 8.6 ± 1.9 μg m^–3^ during the campaign. The OA mass for each ambient particle sample was calculated based on HR-ToF-AMS measured masses of sulfate, inorganic nitrate, chloride, and ammonium. The masses of black carbon and mineral dust and other compositions were assumed to be 5% of the total mass. For more information regarding the sampling times, conditions and masses, see Table S3.

### 2.3 QCM Mass Measurement during OA Dark and Photolytic Aging

Within 10 min of the collection of laboratory SOA or ambient particles, the particle-laden sensor was mounted into a humidity- and temperature-controlled flow cell equipped with a sapphire window (Q-sense QWM401), and the real-time mass under different humidity and UV irradiance conditions was monitored using a high-sensitivity quartz crystal microbalance with dissipation (QCM-D, Q-sense Analyzer). The schematic diagram of the QCM apparatus is depicted in Figure S2. The QCM technique has been previously applied to laboratory-generated OA samples to characterize their volatility,^50^ water diffusivity,^8^ hygroscopicity,^51^ and photolytic mass loss.^19,38^ The QCM operates on the principle that changes in the resonant frequency (Δ*f*) of the quartz crystal are proportional, through a sensitivity factor (ζ), to changes in mass (Δ*m*) on the sensor. This relationship is expressed by the equation Δ*m* = −ζΔf. To ensure accuracy, six different frequency overtones were cross-verified, as shown in Figure S3. By performing sensor cleaning after each experiment using methanol and deionized water, the baseline of a blank sensor can be restored with a frequency error of 50 Hz or mass error of 0.75 μg. In all SOA experiments, the collected mass was controlled to be within the range of 20.0 ± 8.0 μg, minimizing variations across cases. For ambient particles, the collected mass was lower due to lower particle concentration in the ambient atmosphere. The information on collected SOA and ambient particle mass loadings and corresponding experimental conditions is outlined in Table S4. UV lights emitted from two different lamps at wavelengths of 300 nm (UVB) and 345 nm (UVA) can penetrate the optical window of the flow cell and were used to conduct the photolytic aging experiments. Photon fluxes emitted by the two UV lamps are shown in Figure S4. The irradiance applied was approximately an order of magnitude higher than typical ambient conditions to mimic photolytic aging equivalent to multiple days of photolysis in the atmosphere. The temperature of the QCM sensor was controlled at 294.15 ± 0.02 K to allow for isothermal photolytic aging.

To evaluate potential light attenuation through particle film, we calculated the deposited film masses, film thicknesses, and light attenuation ratios for QCM samples in Table S5. The SOA film thicknesses on QCM crystals ranged from 100 to 300 nm. Even at 300 nm wavelength, where SOA absorption is stronger than at 345 nm, the light attenuation remained negligible (<1%) because of the relatively weak absorption of these SOA types. For ambient particles, the film thicknesses were 25–100 nm, and even under the worst estimate using the highest mass absorption coefficient, the light transmission through the film remained >70%. Therefore, no attenuation corrections were applied.

### 2.4 Volatility Measurement during OA Dark and Photolytic Aging

Mass loss rates (evaporation rates) of the deposited particle film were derived as the first derivative of the measured real-time masses. To effectively measure volatility, two equilibrium conditions must be examined: gas-phase diffusion equilibrium and particle-phase diffusion equilibrium. To confirm that the gas-phase diffusion equilibrium was established within the flow cell, we have conducted a flow rate test as shown in Figure S5. During the evaporation of the SOA that contains S/IVOCs, the SOA mass loss under dark conditions would be limited by gas flow rate if film–gas phase equilibrium was established. Our test experiments showed that evaporation mass losses were proportional to the flow rates ranging from 3 to 30 cm^3^ min^–1^, indicating that gas-phase diffusion was sufficiently fast so that the headspace of the flow cell was saturated with the composition of the film surface.^50^ We also justified a rapid liquid-like diffusion equilibrium for fresh SOA examined in this study by observing the evaporation rate under dark conditions at different relative humidity (RH) levels. Since water can act as a plasticizer and increase the diffusivity of the SOA material,^8,50^ if the evaporation was kinetically limited by the in-film diffusion, we would have observed continuously enhanced evaporation mass losses with increased RH. In our dark experiments, for both SOA, evaporation mass losses showed no difference between dry and up to ∼60% RH, implying that the two investigated SOA types are liquid-like and the diffusion process in the film does not impose a significant kinetic limitation on evaporation. Therefore, the measured mass loss rate by QCM was proportional to the volatility of the SOA thin film, i.e.

1 $$C^{*}=\frac{dm}{Qdt}$$

with a sensitivity factor (ζ) of 17.7 ng cm^–2^ Hz^–1^ (provided by crystal vendor), mass changes on the order of 1 ng could be detected in QCM. This mass change corresponded to a vapor mass concentration (volatility) of 33 μg m^–3^ under the experimental conditions employed (*Q* = 30 cm^3^ min^–1^) assuming Δ*t* = 1 min. In dark aging experiments, the flow cell was purged with zero air in the absence of light, and the gradual mass losses were attributed to the evaporation of compounds that have semi- or intermediate-volatility between 10^2^ to 10^5^ μg m^–3^. The long-term mass loss was used to retrieve the volatility distribution of fresh SOA. For photolytic aging experiments, the lamp was periodically turned on and off at 15 min intervals, allowing for the dynamic measurement of the intrinsic volatility of OA, i.e., the vapor mass concentration within the dark periods.

### 2.5 Hygroscopicity Measurement during OA Dark and Photolytic Aging under Humid Conditions

The flow conditions were switched between dry and humidified states at 15 min intervals to enable the measurement of particle dry mass and absorbed water mass, allowing the real-time measurement of hygroscopicity with a resolution of 30 min. Evaporation of the dry mass baseline during this period was accounted for using linear interpolation of the dry mass measurements taken before and after each humidified period. During aging experiments, a constant RH was applied periodically throughout the experiment. In separate hygroscopicity (κ) measurement experiments before and after the aging, different RH levels were scanned to measure the RH dependence of hygroscopicity. The hygroscopicity of the film was calculated using the following equation

2 $$\kappa =\left(\frac{100}{RH\;\left(\%\right)}-1\right)\frac{m_{water}\rho_{dry\;particle}}{\rho_{water}m_{dry\;particle}}$$

The density of OA during aging was assumed to be constant. Note that the relative error in density prediction does not propagate to the relative change in κ. For photolytic aging experiments conducted under humid conditions, the UV light was only turned on during the humid periods, i.e., the particles were irradiated while the flow was humidified in a cofrequency manner, allowing real-time hygroscopicity measurement. The typical frequency profiles for different experiment conditions are provided in Figure S6. There was a slight temperature disturbance induced by irradiance, resulting in a direct frequency offset and an indirect RH adjustment. Such disturbance was corrected to ensure accurate determination of hygroscopicity (Figure S7). The adsorption of water vapor onto the surface of the sensor was found to be 2 orders lower than the absorption (Figure S8).

### 2.6 Nanospray Desorption Electrospray Ionization High-Resolution Mass Spectrometry Measurement

The quartz crystal sensors, prepared in the same way as those used for photolytic aging experiments and loaded with fresh and photolytic aged SOA samples were stored under −18 °C until nano-DESI HRMS measurements. The fresh samples may experience evaporation comparable to that observed in dark aging experiments under dry conditions. The design and implementation of the nano-DESI source can be found elsewhere.^52,53^ Here, all nano-DESI experiments were coupled with a high-resolution LTQ Velos Orbitrap mass spectrometer (Thermo Scientific, Waltham) in negative ion mode. All samples were analyzed by MS1 (*m*/*z* 100–1000) with a mass resolution of 100 000 (unitless) at *m*/*z* 400. The maximum ion injection time was set to 500 ms to reach an automatic gain control (AGC) target of 10^6^. The MS inlet capillary was maintained at 275 °C for all analyzes. The nano-DESI assembly was scanned along the XY plane of the substrate at 75 μm/s and 100 MS1 scans were collected for each sample which were then averaged in Xcalibur (Thermo Scientific) and exported as a *.csv* peak list (5 decimal points per *m*/*z*). Centroided peak lists were subsequently processed via MFAssignR, an open-source molecular formula (MF) assignment software package. Since the SOA samples here were generated under NO_*x*_-free conditions, final MF assignments for the collected SOA samples were limited to the form of C_*x*_H_*y*_O_*z*_ between *m*/*z* 100 and 1000 with restrictions: 0.3 ≤ H/C ≤ 3; O/C ≤ 2.5; −20 ≤ DBE-O ≤ 25 (DBE-O: double bond equivalents minus oxygen count). Blank subtractions were conducted, and final assigned formulas were manually inspected and cleaned for outliers. For Nano-DESI HRMS mass spectra-based volatility prediction, we followed Li et al.^54^

3 $$\log_{10}C_{0}=\left(n_{C}^{0}-n_{C}\right)b_{C}-n_{O}b_{O}-2\frac{n_{O}n_{C}}{n_{C}+n_{O}}b_{CO}$$

where *n*_C_^0^ = 22.66, *b*_C_ = 0.4481, *b*_O_ = 1.656 and *b*_CO_ = −0.779.

### 2.7 Photolytic Rate Conversion from Laboratory Lamps to Solar Irradiance

The product of absorption cross-section and quantum yield σ_Φ_(λ) = σ­(λ)­Φ­(λ) (unit: photon^–1^ cm^2^) values for limonene SOA, isoprene SOA, and ambient OA under humid conditions were calculated by normalizing the model-derived photolysis rates at 300 and 345 nm to a unit photon flux corresponding to the irradiation of laboratory lamps. The photon flux for each experiment was determined based on the frequency offset caused by light (Figure S9). We assume that σ_Φ_(λ) at a logarithmic scale decreased linearly as a function of wavelength. By integrating the product of σ_Φ_(λ) and solar photon flux between 280 and 370 nm, the reaction rate of OA photolytic aging under ambient solar radiation was calculated. Here, solar photon flux from the standard solar spectra was used. 370 nm was roughly the upper limit of 345 nm lamp we used in the laboratory and also the upper limit of limonene SOA photoreactivity. Assuming 370 nm as the cutoff wavelength above which no photolytic reactions occur represents a lower estimate for the ambient photolysis rate.

## 3 Results

### 3.1 SOA Mass Loss

Figure 1 shows the temporal profiles of mass fraction remaining for SOA material derived from isoprene photooxidation (Figure 1a,b) and limonene ozonolysis (Figure 1c,d), representing biogenic OA. During dark aging, both SOA exhibited 20–30% mass decay over 1–2 days purged by zero air, indicating the presence of a significant fraction of S/IVOCs within the organic film.^50^ The evaporation behavior of isoprene-derived SOA showed no discernible difference between dry and humid (∼60% RH) conditions. Similarly, the evaporation of limonene-derived SOA exhibited no enhanced mass loss below 80% RH (Figure S10). Previous studies reported that kinetic diffusion limitations for anthropogenic SOA derived from aromatics disappeared at RH above 20–40%.^8^ These results suggest that the viscosities of both SOA investigated here were low even under dry conditions and that particle evaporation was not limited by diffusion. The dark mass loss rate of limonene-derived SOA increased at RH above 80%, and the mass fraction remaining was lower compared to dry conditions. Such a high transition RH is likely associated with water-promoted reactions rather than the plasticizer effect that typically occurs at lower RH.^8,50^ Water can enhance evaporation by promoting the decomposition of oligomers and increasing SOA volatility. Hydroperoxide dimers in α-pinene ozonolysis SOA were found to decompose into monomers, increasing SOA volatility and evaporation rates at similar high RH.^55^ The different water impacts between the two SOA types might be attributed to their chemical compositions, particularly their functional groups.

**Figure 1:**
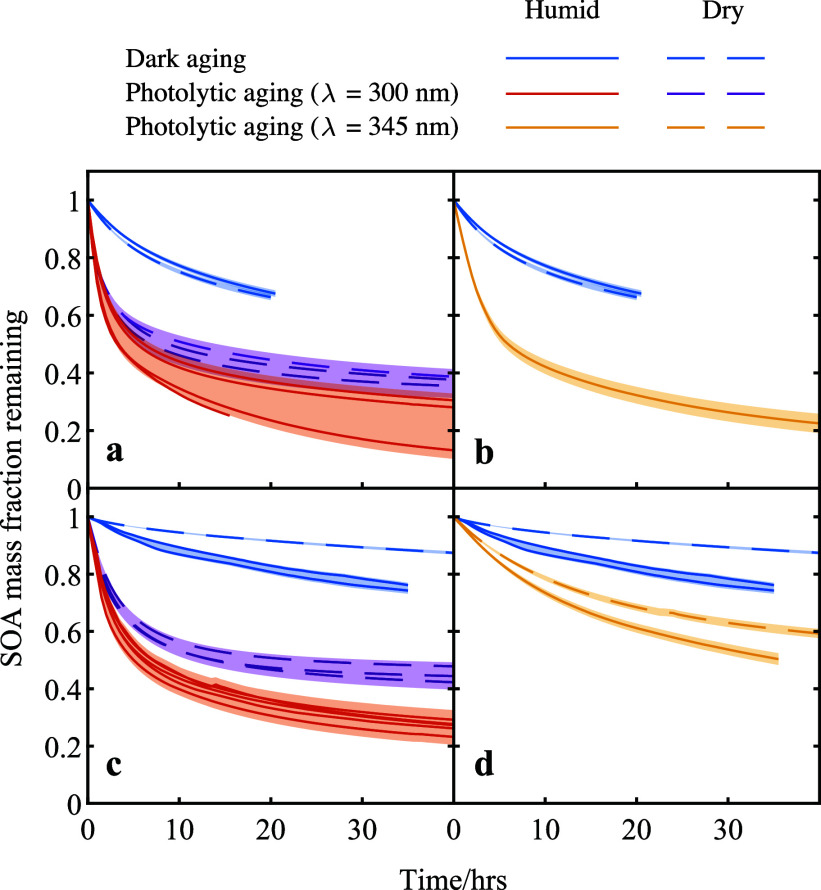
Temporal decay of the mass fraction for isoprene photooxidation SOA and limonene ozonolysis SOA during dark and photolytic aging under varying RH in a QCM flow cell. The upper panels (a,b) show the temporal decay of mass fraction for isoprene photooxidation SOA, while the lower panels (c,d) display the same profiles for limonene ozonolysis SOA. The aging processes include dark aging, photolytic aging with 300 nm light (a,c), and with 345 nm light (b,d). These experiments were conducted at different RH levels in zero air. Under humid conditions, the isoprene SOA experiments were conducted at approximately 60% RH, while the limonene SOA experiments were at around 80% RH. An uncertainty of 0.75 μg was used for a single measurement, and for multiple measurements, a collective uncertainty is shown as the shaded area. The time resolution for plotting is 30 min.

In the photolytic aging experiments, we examined the influences of photolysis wavelength, RH, and the surrounding gas environment. SOA mass losses during photolytic aging were faster and more extensive than during dark aging, suggesting the importance of photolytic mass loss under atmospherically relevant UV wavelengths. Notably, a residual photorecalcitrant fraction was observed in all photolytic aging experiments. The mass loss rates under 300 nm light were higher than those under 345 nm light for both types of SOA when normalized to the same photon flux (Figure S11). Both SOA showed stronger absorption at 300 nm than at 345 nm, and the higher photon energy at 300 nm could contribute to a higher quantum yield.^56^

Regarding the effect of RH, photolytic mass loss fractions were higher under humid conditions for both types of SOA after 40 h of UV irradiation, indicating that aerosol water can enhance photolytic mass losses. Previous studies have found that higher RH promotes SOA photolysis,^16,38^ although inhibiting effects have been reported elsewhere.^23^ Several plausible mechanisms could explain how aerosol water affects photolytic mass loss rates, including matrix effects related to viscosity,^57,58^ viscosity-induced diffusion limitations, or direct water participation in photolytic reactions. In this study, diffusion-limited evaporation was not observed for either type of fresh SOA during dark evaporation. We hypothesize that water can facilitate fragmentation or induce alternative reactions (see SOA Chemical Composition Change).

The presence of O_2_ also had a substantial impact on photolytic reactions. Compared with photolytic aging experiments conducted in air, the photolytic aging in N_2_ showed a higher initial mass loss rate for the first 1–2 h, but a lower overall mass loss fraction afterward (Figure S12). In a test experiment, we replaced N_2_ with air after long-term irradiation and observed an elevated photolytic mass loss rate (Figure S13). These results imply that photosensitization-mediated secondary processes might play an important role during photolytic aging depending on the availability of O_2_. For example, triplet excited states can act as precursors for singlet oxygen, superoxide, hydroperoxyl radicals, and hydroxyl radicals upon reactions with O_2_. The unavailability of O_2_ prevents the formation of these oxidants, which might explain the inhibited mass losses in N_2_.

### 3.2 SOA Volatility Evolution

Figure 2a shows the mass loss rates of isoprene SOA in dark and with 300 nm UV light turned on and off at 15 min intervals under dry conditions. Upon irradiation, the mass loss rate increased by an order of magnitude compared to dark evaporation, indicating that UV light can directly trigger fragmentation reactions and produce gases, leading to rapid mass loss. However, even after the light was turned off following 15 min of irradiation, the mass loss rate remained elevated compared with dark aging under both dry and humid conditions (Figure S14). This observation suggests that photolytic aging produces more volatile S/IVOCs, for which evaporation continues after irradiation and is kinetically limited by their volatility. These S/IVOCs (termed as more volatile organic species, MVOS, in this study), quantified by the QCM, had volatilities between 100 and 10^5^ μg m^–3^ (see Text S1). Species with volatilities ≤10 μg m^–3^ were termed as less volatile organic species (LVOS) here. The release of MVOS into the gas phase in the QCM flow cell was kinetically limited by their volatility under both dry and humid conditions, which explains the extended evaporation after the light was turned off. The rapid decrease of mass loss rates during each light-off periods resulted from the evaporation of relatively high volatility species (∼10^5^ μg m^–3^). We consider photolytic aging of particles to involve two distinct processes driven by photoinduced reactions. The first process corresponds to the production of highly volatile VOCs that evaporate immediately upon irradiation, and their losses gradually decline as the reactants are consumed. These reactions produce volatile species such as carbon monoxide, methane, and small oxidized VOCs (OVOCs), whose evaporation is kinetically limited by the rates of the underlying photochemical reactions. The second process involves the formation of semi- or intermediate-volatility MVOS, whose evaporation increases gradually as photolytic reactions produce and accumulate these products in the particle phase and their release is limited by their partial vapor pressures. Consequently, the overall mass loss rate does not decrease monotonically during each light-on period; instead, it evolves as MVOS production and evaporation progressively approach equilibrium. As photolysis rates decrease with time, evaporation losses become the dominant mass decay process, as indicated by monotonically increasing mass loss rate during each light-on period after 8 h.

**Figure 2:**
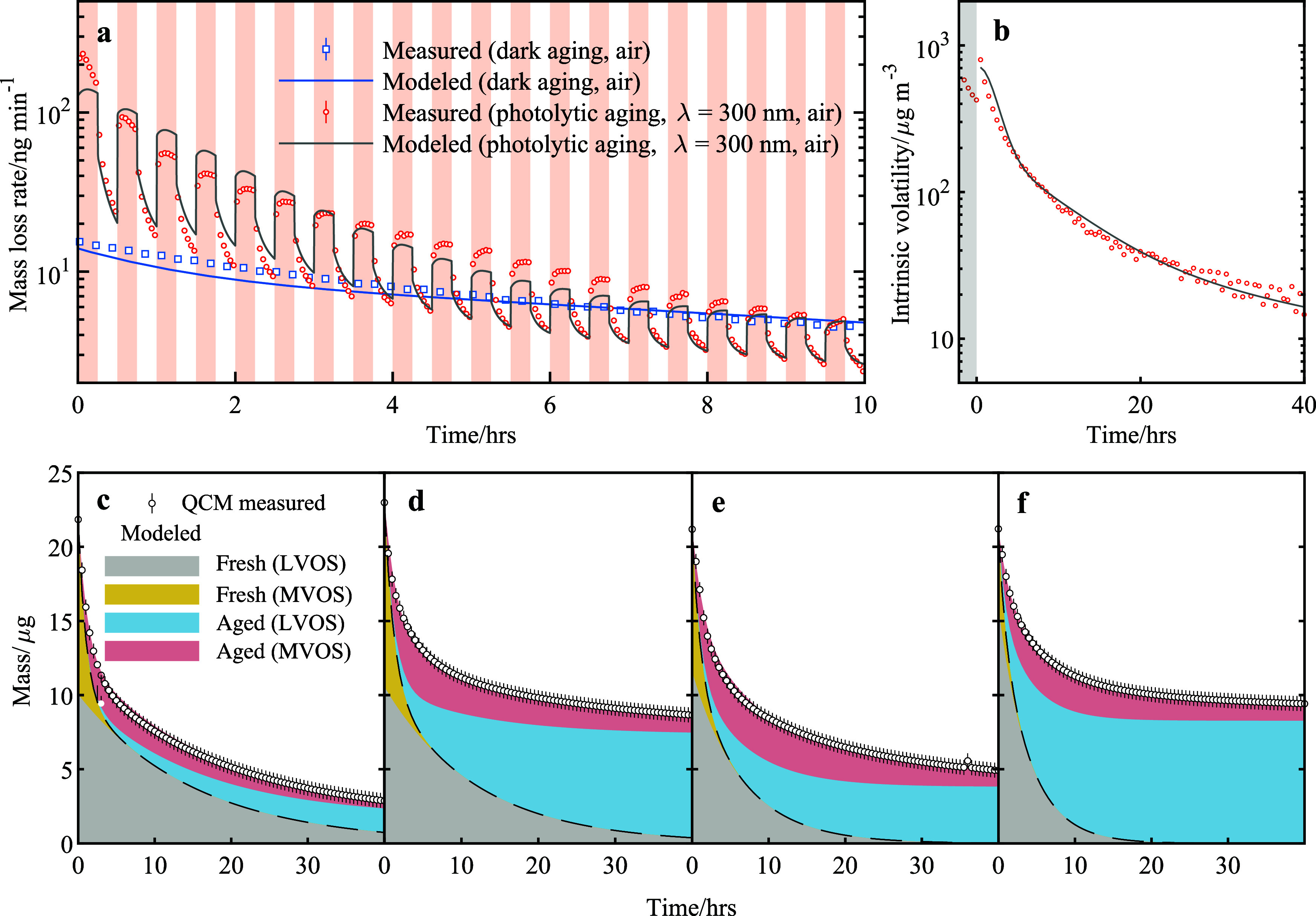
Measured and modeled mass loss rates, intrinsic volatilities and absolute masses of isoprene and limonene SOA during photolytic aging under humid and dry conditions in a QCM flow cell. (a) Comparison of measured and modeled mass loss rates for isoprene SOA during dark aging and photolytic aging with 300 nm light under dry conditions. The temporal resolution is 2.5 min. Pink shading represents periods of irradiation. The time axis denotes the relative time since the first light-on. (b) Comparison of measured and modeled intrinsic volatilities of isoprene SOA under dry conditions. The shown intrinsic volatilities correspond to the last 2.5 min of each light-off period. Intrinsic volatility was modeled using a volatility box model that incorporates photolytic volatility transformation. Further model details are provided in Text S1. (c–f) Measured and modeled absolute masses for isoprene and limonene SOA under both humid and dry conditions. The modeled mass is segregated into fresh MVOS (more volatile organic species), fresh LVOS (less volatile organic species), aged MVOS, and aged LVOS.

To examine long-term SOA volatility transformations during photolytic aging, we depict the volatility measured during the last 150 s of each light-off period for isoprene SOA and limonene SOA under both dry and humid conditions (Figure 2b and Figure S14). This value represents the lowest volatility prior to each light-on period and provides robust evidence that photolytic aging produces MVOS and increases SOA volatility at the initial stage of irradiation. These results highlight the importance of considering the volatility and evaporation of photolytic aging products, as the observed mass decay may appear slower than the underlying photolysis rates. Moreover, the evaporation and partitioning of S/IVOCs can be strongly influenced by the OA mass concentration, which is significantly lower in ambient atmosphere than in laboratory experiments. Constrained by the observed long-term volatility evolution, the modeled underlying SOA compositions segregated by volatility are shown in Figure 2c–f (see Modeling).

### 3.3 SOA Hygroscopicity Evolution

The evolutions of the hygroscopicity parameter (κ) as a function of mass fraction remaining under humid conditions are depicted in Figure 3 for isoprene photooxidation SOA (Figure 3a) and limonene ozonolysis SOA (Figure 3b). During dark aging, both isoprene SOA and limonene SOA exhibited a minor increase in κ. In contrast, these two types of SOA showed distinct κ changes during photolytic aging, associated with their initial chemical compositions. For isoprene SOA photolytic aging in air, κ remained unchanged until the remaining mass fraction dropped below 0.3, after which κ sharply decreased with further mass decay. In contrast, limonene SOA photolytic aging under 300 nm light in air exhibited a rapid κ increase with mass loss, followed by a slight κ reduction at later stages. Consistent κ trends were observed across replications (Figure S15) and similar κ changes were observed after aging across the entire RH range (Figure S16). On average, during photolytic aging under humid conditions with 300 nm light, isoprene SOA κ changed from 0.18 ± 0.02 to 0.16 ± 0.02 while limonene SOA κ increased from 0.06 ± 0.00 to 0.11 ± 0.01. Although temporal information was not available, dry photolytic aging resulted in a greater decrease in isoprene SOA κ from 0.18 ± 0.02 to 0.14 ± 0.02, while limonene SOA κ remained unchanged at 0.06 ± 0.00 (Table S6). For photolytic aging in N_2_ with 300 nm light, κ values at 45% mass fraction remaining were 0.15 ± 0.01 and 0.06 ± 0.00 for isoprene SOA and limonene SOA, respectively, substantially lower than 0.20 ± 0.01 and 0.10 ± 0.01 in air. Those results indicate that photolysis-induced secondary in-particle reactions vary depending on the gas environment.

**Figure 3:**
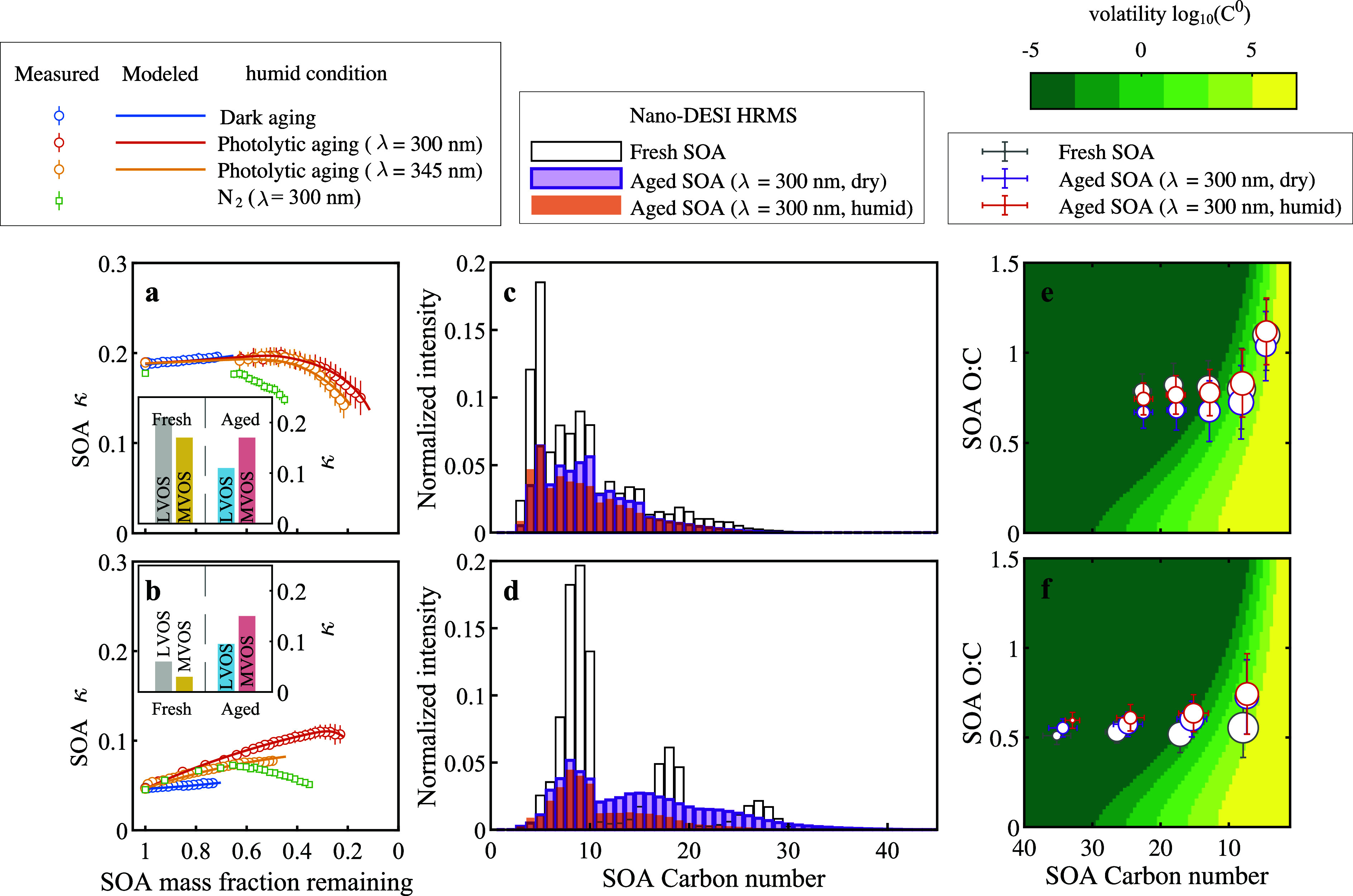
Hygroscopicity (κ) evolution and chemical composition transformation of SOA. The left panels (a,b) show the hygroscopicity (κ) evolution as a function of mass fraction remaining for isoprene photooxidation SOA (a) and limonene ozonolysis SOA (b) during dark and photolytic aging under different lamp wavelengths and gas compositions in a QCM flow cell. Data points represent binned averages at 2% mass fraction intervals. To model κ evolution in zero air, each segregated composition, i.e., fresh MVOS, fresh LVOS, aged MVOS, and aged LVOS, was assigned a unique κ value. Determined parametrization is shown in the insets. The middle panels (c,d) show normalized Nano-DESI HRMS signal intensities by carbon number for fresh and 300 nm UV-aged samples under humid and dry conditions for isoprene photooxidation SOA (c) and limonene ozonolysis SOA (d). The total signal after aging was further scaled based on QCM-measured mass fraction remaining. The right panels (e,f) show the O/C ratios segregated by a monomer unit (carbon number = 5 for isoprene SOA, e and 10 for limonene SOA, f). Volatility was calculated based on carbon number and O/C following Li et al.^54^

### 3.4 SOA Chemical Composition Changes

To relate photolysis-induced changes in SOA volatility and hygroscopicity to associated changes in SOA chemical composition, we conducted Nano-DESI HRMS measurements directly on QCM crystal sensors for both fresh samples and 300 nm UV-aged samples under dry and humid conditions. Figure 3c,d shows the signal intensities of molecules at each carbon number that are normalized by the total signal intensity for fresh and aged isoprene photooxidation SOA (Figure 3c) and limonene ozonolysis SOA (Figure 3d). Figure 3 also shows the signal intensity weighted oxygen-to-carbon (O/C) ratios segregated by monomer unit (carbon number = 5 for isoprene SOA in Figure 3e and 10 for limonene SOA in Figure 3f). The colored background shows volatility calculated based on carbon number and O/C ratio following Li et al.^54^

Various high-volatility C1–C3 vapors, including carbon monoxide, methane, ethene, and small OVOCs such as formaldehyde, formic acid, acetaldehyde, acetone, acetic acid, and methanol, have been identified as dominant products of OA photolytic aging.^17,19,22,27,28,39,59–61^ Although direct vapor measurements were not available here, Nano-DESI HRMS results for limonene SOA provided consistent insights for the particle-phase remaining after aging. Fresh limonene SOA showed distinct monomer and oligomer patterns (Figure 3d). After photolytic aging under humid conditions, the average molecular weight of dimers decreased from 17.2 to 14.9, and that of trimers decreased from 26.4 to 23.4. Under dry conditions, these values changed to 15.3 and 24.3, respectively (Figure 3f). These results agree with the formation of C1–C3 vapors as dominant products from photolytic reactions. In contrast, the monomers only showed an average reduction in carbon number of about 0.5. Following cleavage of the original carbon skeleton, the fate of the remaining moiety depends on its volatility. The remaining moiety of oligomers with very low volatility can still reside in the particle phase, while for many monomers, the loss of 1–3 carbons might result in the formation of S/IVOCs or even vapors. Consequently, the observed carbon number decrease of particle phase monomers is further regulated by an upper volatility bound. As shown in Figures 3e, f, and S17, S18, the carbon number vs O/C spaces of both fresh and aged SOA are well constrained by volatility on the low carbon number and low O/C side. These results agree with the observed temporary volatility increase (see SOA Volatility Evolution) and underscore the necessity of considering volatility transformation and partitioning behavior in photolytic aging kinetics.

The measured κ changes can be explained by the combined effects of O/C ratio changes and molecular weight changes. The O/C ratio has been found to correlate positively with κ of OA samples previously,^62–64^ although such a relationship may be mediated through solubility.^65–68^ On the other hand, κ decreased with increasing molecular weight for fully dissolved organic compounds or organic mixtures.^66,69,70^ For isoprene SOA, the average molecular weight changed insignificantly (<3%) after photolytic aging under dry and humid conditions (Table S6), agreeing with previous research.^21,40^ The average O/C of isoprene SOA remained unchanged under humid conditions (from 0.90 to 0.88) but decreased to 0.77 under dry conditions. A decrease in O/C was also observed during isoprene SOA photolytic aging in previous chamber study.^23^ Since mass loss under humid conditions was higher than under dry conditions, the unchanged O/C under humid conditions suggests that water could induce different in-particle reactions that lead to additional oxygen incorporation and compensate for oxygen loss. These measured O/C changes agree with the minor κ decrease under humid conditions and the larger κ decrease under dry conditions.

For limonene SOA, distinct changes in chemical composition were found between photolytic aging under humid and dry conditions. The different effects of water on photolytic reactions of isoprene and limonene SOA can be attributed to their contrasting interactions with water, as also demonstrated in dark aging experiments. Under humid conditions, molecules in limonene SOA underwent water-induced decomposition prior to photoreactions, and Nano-DESI HRMS results revealed a strong reduction in oligomer signals after photolytic aging. In contrast, under dry conditions, molecules larger than the original trimers were detected, and the signal fraction of molecules with carbon number >30 increased from 0.5% to 2.9%, indicating oligomerization. Limonene ozonolysis SOA was less oxidized and contained more carbonyl and hydroxyl functional groups, which can promote oligomerization pathways such as esterification. In contrast, isoprene SOA was highly oxidized and enriched in carboxylic acid groups, which are less favorable for oligomerization.^21^ Moreover, previous studies have reported that oxidation during dry photolytic aging gradually increases the viscosity of limonene SOA, creating diffusion-limited conditions that might favor oligomerization.^40^ These results are consistent with previous studies showing no oligomer formation through photolytic reactions of limonene SOA in bulk water,^26^ but significant oligomerization under dry particle conditions.^40^ Meanwhile, the measured limonene SOA O/C ratio increased from 0.53 to 0.69 under humid conditions and to 0.64 under dry conditions, similar to previous research for monoterpene ozonolysis SOA.^21,38,40^ Following the combined chemical composition changes, κ of limonene SOA showed a substantial increase under humid conditions in contrast to no change under dry conditions (Table S6), as O/C increase effect under dry conditions was offset by a concurrent increase in average molecular weight from 267 to 338 Da, which acts to decrease κ.

The different directions of O/C ratio changes observed for the two SOA types studied can be related to their initial oxidation states. The O/C change reflects a combination of oxygen loss during carbon skeleton cleavage and oxygen addition in air. Fresh isoprene SOA was highly oxidized and more prone to losing oxygen atoms during carbon skeleton cleavage. In contrast, for fresh limonene SOA with a relatively lower O/C ratio (0.53), photolytic oxygen addition was more likely to surpass oxygen loss and to increase O/C. The O/C ratios for photolytically aged particles under humid conditions were higher than those under dry conditions, possibly because secondary reactions involving water promoted additional oxygen incorporation and generated new highly oxygenated photolytic products. In N_2_, where oxygen was unavailable, both SOA exhibited lower κ values during photolytic aging.

The average molecular weights of SOA either remained unchanged or increased after photolytic aging (Table S6), which may seem counterintuitive. This can be explained by two factors. First, particle-phase species are regulated by volatility; as S/IVOCs and vapors evaporate, the expected decrease in molecular size cannot be observed in particle phase. Second, as shown in Figure 3c, d, monomers exhibited the greatest loss, resulting in higher oligomer-to-monomer ratios. In some cases such as limonene SOA aged under dry conditions, oligomerization played a significant role, leading to increases in both the oligomer-to-monomer ratio and the average molecular weight.

### 3.5 Ambient OA Photolytic Aging

Photolytic aging experiments were performed on ambient fine particle samples collected in Atlanta during summer. High OA mass fractions (0.67–0.84) were determined by HR-ToF-AMS measurements (Table S3). Major OA factors, including more-oxidized oxygenated organic aerosol (MO-OOA), less-oxidized OOA (LO-OOA), and isoprene-derived OA, together constituted 0.77–0.89 of the OA mass, implying that most of the OA mass was SOA. No significant mass loss was observed after 10 h of dark aging (Figure 4a), indicating that the ambient OA was predominantly low-volatility species, as expected based on the gas–particle partitioning under low OA mass concentrations. 40–50% of the ambient OA mass was depleted after 40 h of photolytic aging with either 300 or 345 nm light under humid conditions (RH ∼ 60%), the latter representing the most atmospherically relevant conditions. In contrast, mass loss was around 10% under dry conditions with 345 nm light. Water played multiple roles in facilitating photolytic mass loss. For limonene SOA, water promoted oligomer-to-monomer fragmentation prior to photoreactions. For isoprene SOA, water could induce alternative photolytic pathways involving additional oxygen incorporation. Water might also act as a plasticizer, enhancing particle-phase diffusion and thereby influencing secondary reactions and the evaporation of photolytically produced MVOS. The higher photolytic mass losses observed under humid conditions for ambient particles may result from a combination of these factors. We conducted a control experiment using reduced-intensity 345 nm irradiance (13.3 W m^–2^, approximately an order of magnitude lower than the photon fluxes typically used in other experiments, but still comparable to ambient irradiance) with similar particle mass loadings. After normalizing by UV intensity, the reduced-intensity experiment showed a faster mass loss but a similar mass loss fraction (Figure S19). Under higher irradiance, the faster photolytic reaction rates cause MVOS to accumulate to higher mass fractions until photolytic production is balanced by evaporation, which reduces the apparent mass decay because more mass initially remains in the particle phase. The results suggest that converting laboratory results using UV dose alone may underestimate photolytic mass loss rates, and that mass loss rates in real atmosphere can be higher with longer time to evaporate S/IVOCs.

**Figure 4:**
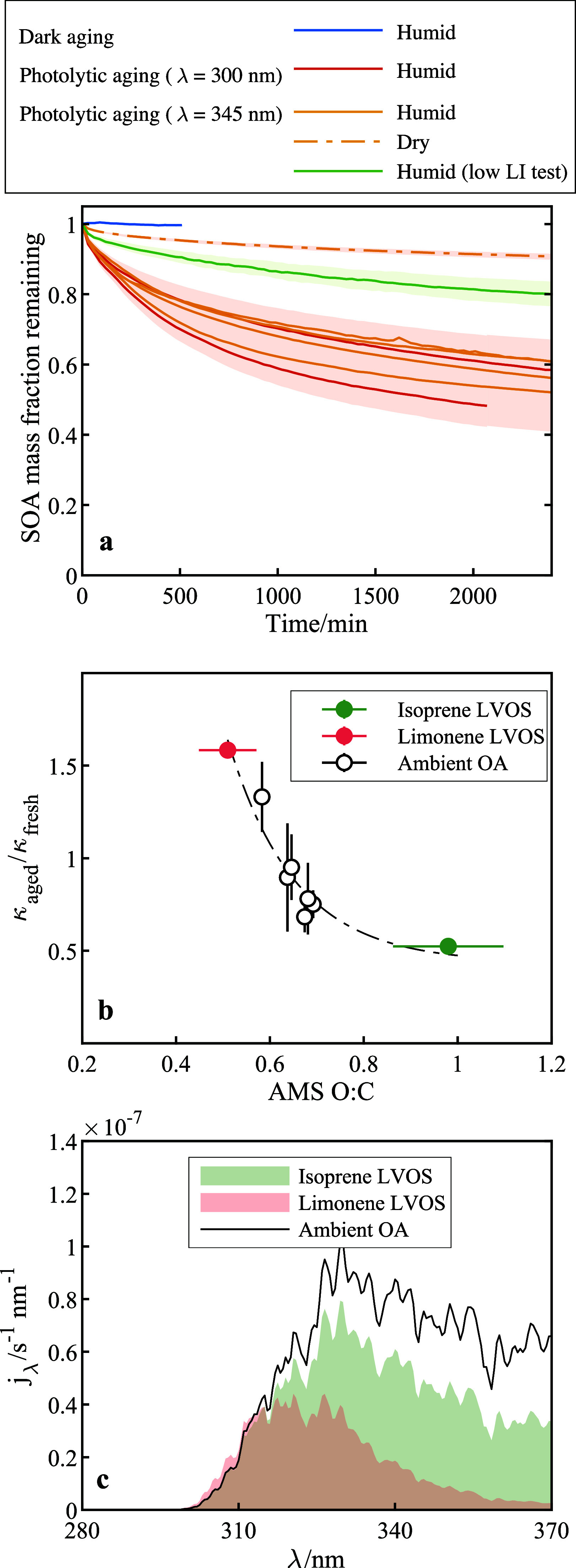
Analysis of photolytic aging processes in ambient organic aerosols. (a) Temporal decay of mass fraction for ambient OA during dark and photolytic aging under varying RH in a QCM flow cell in zero air. A control experiment was conducted using low-intensity 345 nm irradiance (13.3 W m^–2^, an order of magnitude lower than typical irradiance). The shaded area represents the mass-induced uncertainty. For photolytic aging under humid conditions with typical irradiance, uncertainties for all mass decay curves are merged. (b) Hygroscopicity change (κ_aged_/κ_fresh_) for isoprene SOA LVOS, limonene SOA LVOS, and ambient OA as a function of the O/C ratios before aging. The hygroscopicity changes for SOA were determined using volatility box model. For ambient OA, the hygroscopicity changes were obtained from the measured values before and after ∼40 h of photolytic aging. The dashed dot line is shown to guide the eye. (c) Wavelength-dependent ambient photolysis rates (*j*_λ, amb_) under standard solar radiation for isoprene SOA LVOS, limonene SOA LVOS, and less photoreactive ambient OA LVOS under humid conditions.

The κ change of ambient OA after 40 h of photolytic aging was also determined and the mass-dependent profiles are provided in Figure S20. Ammonium sulfate was assumed to have minor contributions to particle water absorption at ∼60% RH. Nitrate contributed negligible mass (Table S3). Figure 4b demonstrates that the photolytic κ change (κ_aged_/κ_fresh_) under humid conditions for both laboratory SOA LVOS and ambient OA followed a consistent trend dependent on the AMS-measured initial O/C ratio of OA. Photolytic aging increased the O/C ratios of less oxidized SOA, such as monoterpene ozonolysis SOA, through oxidation and functionalization, but decreased the O/C ratios of highly oxidized SOA, such as isoprene SOA. Because changes in average molecular weight were minor, the direction of O/C ratio changes were consistent with the changes in κ. Consequently, photolytic aging under humid conditions increased κ for less oxidized OA but decreased κ for more oxidized OA, resulting in a negative relationship between κ change and the initial O/C ratio of OA.

### 3.6 Modeling

We developed a volatility box model to predict the observed mass decay, volatility changes and hygroscopicity evolution within a unified framework during photolytic aging. Details of the box model are provided in Text S1. In brief, the model categorizes SOA species into decadal volatility bins, and first-order photolytic reactions are incorporated by parametrizing the transformation of these volatility bins into products having different volatilities. To constrain the model, we first optimized a volatility distribution with four volatility bins (*C* ^0^ ≤ 10, 10^2^, 10^3^, 10^4^ μg m^–3^) for fresh SOA that reproduced the observed dark evaporation behavior (Figure 2a, S21, Table S7). Our results agree with reported limonene SOA volatility distributions derived in chamber under dry conditions.^71^ Approximately 54% of the collected mass of isoprene SOA was MVOS. For limonene SOA, the fraction of MVOS increased from 25% under dry conditions to 45% under humid conditions.

The fresh SOA species (fresh MVOS + LVOS) were then allowed to evaporate and photolyze competitively in a QCM flow cell during photolytic aging. In our final model, we allowed fresh LVOS and fresh MVOS to have different photolysis rates and assumed the photolytic products span from LVOS to MVOS and vapors (*C* ^0^ ≥ 10^6^ μg m^–3^). Both MVOS formation and volatility-dependent photolysis rates are necessary to explain the observed volatility evolution. We measured dark mass loss rates before and after the first light-on period under both dry and humid conditions and found substantially enhanced dark evaporation after photolytic aging, which verified the MVOS formation (Figures 2a,b, S14, S22). In a control experiment, SOA was allowed to evaporate in the dark for 20 h before the light was turned on. We found that the normalized mass loss rates (photolysis rates) decreased by 40% to 70% after evaporation, confirming that the evaporated SOA fraction (i.e., MVOS) exhibited higher photolysis rates.^61^ These two factors were then simultaneously optimized in our photolytic volatility transformation model and a higher photolysis rate for fresh MVOS than that of fresh LVOS explained the decreased photolysis rates well after fresh MVOS evaporation (Table S8). The optimized parametrization for the final model of SOA photolytic aging can be found in Table S9. The modeled temporal profiles of the underlying composition, categorized as fresh LVOS, fresh MVOS, aged LVOS, and aged MVOS are shown in Figure 2c–f. Fresh MVOS were rapidly consumed during photolytic aging, accompanied by the formation of aged MVOS that had higher volatility than fresh MVOS on average. The photolytic loss of fresh LVOS prevailed for a longer time and a fraction of the mass was transformed into aged LVOS residing in the particle phase. Those aged LVOS have previously been termed photorecalcitrant SOA.^20,23,38^

The optimized model was then used to predict κ profiles under different conditions by assigning four κ values to fresh LVOS, fresh MVOS, aged LVOS, and aged MVOS. Good agreement between the measurements and model predictions was found. The composition-specific κ values for isoprene SOA and limonene SOA are shown in the insets of Figure 3a,b, respectively. For isoprene SOA, fresh LVOS had a higher κ (0.21) than fresh MVOS (0.17), consistent with the observed κ changes during dark aging. Aged MVOS had a κ value of 0.17, while aged LVOS had a much lower κ of 0.11. The initial stage of isoprene SOA photolytic aging was characterized by rapid consumption of fresh MVOS and accumulation of aged MVOS, resulting in no net change in κ. However, as fresh LVOS was transformed into aged LVOS (Figure 2e), κ decreased substantially. For limonene SOA, fresh LVOS and fresh MVOS had κ values of 0.06 and 0.03, respectively, while κ values of both aged MVOS (κ = 0.15) and aged LVOS (κ = 0.095) were much higher. Evaporation of the most hydrophilic MVOS can explain the slight κ reduction observed at later stages of photolytic aging under 300 nm light. The κ value of SOA aged under 300 nm light was higher than that observed under 345 nm light at the same mass fraction, because of more accumulation of aged MVOS driven by the higher photolysis rate under 300 nm.

The formation of S/IVOCs had an important influence on hygroscopicity evolution and photolysis kinetics. First, the underlying photolysis rates can be much higher than those calculated by the observed mass decay curves because of the slow evaporation of S/IVOCs. Photolysis rates determined by models with S/IVOCs formation were 1–2.5 times higher than those ignoring S/IVOCs formation (Table S9, S10, S11). The rapid depletion and regeneration of S/IVOCs also depict a more dynamic partitioning nature of SOA during daytime. Previously reported discrepancies between photolytic particle mass loss and released vapor mass might also be explained by S/IVOCs formation. For the two investigated SOA, evaporation of MVOS represented 44–92% of the mass loss of SOA (Table S9). Previous studies combining Proton Transfer Reaction Time-of-Flight Mass Spectrometer (PTR-ToF-MS) and QCM measurements found that the measured vapors represented ∼50% of the SOA mass losses.^19^ However, it should be recognized that total mass is not conserved considering the oxygen addition during SOA aging in air. The “true” vapor mass is obtained by direct vapor measurements, whereas mass changes in substrates or particles represent the “net” vapor mass loss.

### 3.7 Atmospheric Implications

The photolysis rates for limonene, isoprene SOA, and ambient OA under laboratory irradiance were determined by our volatility box model (Text S1). We then calculated the products of absorption cross section and quantum yield (σ_Φ_ values) by normalizing the model-derived photolysis rates at 300 and 345 nm for MVOS and LVOS (Figure S23, Table S12). The σ_Φ_ values for limonene and isoprene SOA MVOS were 15–106% of their corresponding SOA absorption cross sections, suggesting that fresh MVOS signifies the fresh SOA absorptivity and exhibits high photoreactivity. Carbonyls^26,60^ and organic peroxides^25,59^ have been identified as the main chromophores around 300 nm and are responsible for OA photolytic reactions. Previous research has also shown that the initial stage of α-pinene SOA photolytic aging is accompanied by a rapid decrease in absorptivity associated with carbonyls depletion.^21^ We then multiplied the σ_Φ_ values by the solar photon flux to calculate the wavelength-dependent ambient photolysis rate *j*_λ, amb_ assuming that log­(σ_Φ_) decreases linearly with wavelength. Figure 4c shows *j*_λ, amb_ for the less photoreactive compositions of the three types of OA under humid conditions. The dominant wavelength range for photolytic aging in the ambient atmosphere was found to be between 320–350 nm, reflecting a compensation between increasing photon flux and decreasing σ_Φ_ as the wavelength increases. The integrated photolysis rate under standard solar irradiance, denoted as *j*_amb_, was then calculated by integrating *j*_λ, amb_ from 280 to 370 nm. The resulting j_amb_ values and their corresponding lifetimes for different compositions of different OA are summarized in Table S13.

A rapid photolytic decay with lifetimes ranging from 8 to 36 h (0.26–1.13% *j*_NO_2__) was found for the more photoreactive components. The lifetimes determined here agree with previous estimates of aliphatic carbonyl photolysis lifetimes of several hours for limonene ozonolysis SOA.^26,60^ The calculated photolysis rate for isoprene MVOS was 3.0 ± 0.5 × 10^–5^ s^–1^ or 1.00 ± 0.17% *j*_NO_2__, which is only slightly lower than that reported for isoprene SOA in a chamber study (1.5 ± 0.3% *j*_NO_2__).^23^ One difference between chamber experiments and substrate experiments is that photolensing effect can occur within suspended submicron particles, amplifying the photo flux by a factor of 1–3.^72^ We also recognize that photon flux can be doubled by substrate reflection here, therefore the measurements for suspended particles and thin films on substrates are comparable. A slower decay with lifetimes ranging from 80–200 h (0.04–0.12% *j*_NO_2__) was determined for the less photoreactive components, which is still significant compared to the physical lifetime of OA in the atmosphere. LVOS represented 46% of isoprene SOA mass and 55% of limonene SOA mass under humid condition. It turned out that 15% of the ambient OA was rapidly photolyzed with complete mass loss while the remaining 85% photolyzed more slowly, and a relative mass fraction remaining of 60.3 ± 5.2% was determined. This remaining fraction was higher than that determined for isoprene SOA LVOS (34.0 ± 19.1%) and limonene SOA LVOS (40.2 ± 4.7%). This result is expected because the collected particles were already partially photolyzed in ambient air. Moreover, compositions other than SOA might have a higher photolytic mass remaining.

## 4 Discussion

In this study, we showed that photolytic aging increased OA volatility and reduced 40–66% of the low-volatility OA mass with lifetimes ranging from 8 to 200 h (0.04–1.00% *j*_NO_2__) under solar irradiance. Previous modeling studies have applied a lower photolysis rate of 0.02% j_NO2_ yet still found a strong effect of SOA photolytic aging in decreasing the budget and reshaping the spatiotemporal distribution of atmospheric SOA.^4,33,34^ However, these modeling studies applied complete mass loss, which contrasts with the photorecalcitrant fractions found here and in other laboratory studies.^20,23,38^ Therefore, higher photolysis rates combined with remaining photorecalcitrant fractions should be applied in chemical transport models to better represent OA photolytic aging. Notably, we found that photolytic-induced reactions led to the formation of highly volatile vapors that rapidly entered the gas phase, semi- or intermediate-volatility moieties that underwent dynamic partitioning, and photorecalcitrant low-volatility products that resided in particle phase with distinct physicochemical properties. Our findings revealed that the κ of OA could be significantly altered by photolytic aging, and the direction of change followed combined effects of O/C ratio changes and molecular weight changes. The κ change direction and magnitude were influenced by the initial OA O/C ratio, humidity, and gas environment. Photolytic oligomerization could play an important role by increasing average molecular weight and suppressing κ, although the specific conditions favoring oligomerization remain unclear and warrant further investigation.

We developed a volatility kinetic model that incorporates photolytic volatility transformation. Our model reproduces the observed nonmonotonic high-resolution mass decay profiles, including both light-on and light-off periods as continuous constraints, and the κ evolution profiles, highlighting the importance of volatility transformation, particularly the formation of S/IVOCs, during photolytic aging. Our model can be used to predict photolytic aging processes in the ambient atmosphere with better recognition of underlying volatility changes. As an example, the volatility distribution of ambient isoprene-derived organic aerosol was used to simulate photolytic mass loss in ambient atmosphere. Fresh SOA with a volatility distribution from Lopez-Hilfiker et al.^73^ was subjected to solar radiation within our thermodynamic model in ambient particles for 240 solar hours (Text S1). The results show that photolytic aging with a higher initial mass concentration leads to a higher photorecalcitrant fraction because more semivolatile products can reside in the particle phase as a result of partitioning (Figure S24). The evaporation of S/IVOCs might be kinetically limited by particle-phase diffusion under low RH conditions as SOA becomes increasingly viscous during photolytic aging,^40^ in an originally viscous organic matrix,^50^ or when the OA is transported higher up in the atmosphere. Our volatility model offers a basic framework for understanding and predicting the complex dynamics of OA during photolytic aging. Although the current modeling framework does not explicitly include photosensitized reactions, such processes represent a plausible additional pathway for OA aging and are an important topic for future investigation. Broader types of laboratory-generated and ambient OA and the interplay between viscosity and photolytic aging should also be explored in future studies.

## Supplementary Material



## Notes

Additional description of the volatility model; details of film light-attenuation calculations; tabulated experimental details; model results; and supplementary figures illustrating the experimental setup, method validation, and supporting experimental results. S1. Volatility box model S2. QCM film thickness and light attenuation ratio calculations Tables S1–S13 Figures S1–S24 (PDF)
