# Supplementary material for: Dynamic Evolution of Mass and Physical Properties of Atmospheric Organic Aerosol Under Solar Irradiance
Source: Environ Sci Technol. 2026 Feb 18;60(8):6464–76. doi: 10.1021/acs.est.5c16671 (PMC12961944; doi:10.1021/acs.est.5c16671)
Supplement: Supplementary file 1 [file es5c16671_si_001.pdf]

Supplementary Information

Dynamic Evolution of Mass and Physical Properties of Atmospheric Organic Aerosol under Solar Irradiance

by

Bin Bai,[1] Gregory W. Vandergrift,[2] Yutong Liang,[3,4] Yaowei Li,[5] Zezhen Cheng,[2] Yuchen Wang,[3,6] Nara Shin,[1] Frank Keutsch,[5,7,8] Andrew Lambe,[9] Swarup China,[2] Nga Lee Ng,[1,3,10] and Pengfei Liu[1*]

Affiliations:

[1] School of Earth and Atmospheric Sciences, Georgia Institute of Technology, Atlanta, Georgia 30332, United States.

[2] Environmental Molecular Sciences Laboratory, Pacific Northwest National Laboratory, Richland, Washington 99354, United States.

[3] School of Chemical and Biomolecular Engineering, Georgia Institute of Technology, Atlanta, Georgia 30332, United States.

[4] Thrust of Sustainable Energy and Environment, The Hong Kong University of Science and Technology (Guangzhou), Guangzhou, Guangdong 511453, China.

[5] School of Engineering and Applied Sciences, Harvard University, Cambridge, Massachusetts 02138, United States.

[6] College of Environmental Science and Engineering, Hunan University, Changsha, Hunan 410082, China.

[7] Department of Chemistry and Chemical Biology, Harvard University, Cambridge, Massachusetts 02138, United States.

[8] Department of Earth and Planetary Sciences, Harvard University, Cambridge, Massachusetts 02138, United States.

[9] Aerodyne Research, Billerica, Massachusetts 01821, United States.

[10] School of Civil and Environmental Engineering, Georgia Institute of Technology, Atlanta, Georgia 30332, United States.

Supporting information includes 43 pages, including 2 texts, 13 tables and 24 figures in total.

S1. Volatility box model

S2. QCM film thickness and light attenuation ratio calculations

Table S1–13

Figure S1–24

S1. Volatility box model

A box model was developed based on thermodynamic considerations, assuming instant diffusion mixing within the particle film. This assumption should be valid for the investigated two SOA as no diffusion limitations were observed for the evaporation of S/IVOCs under dry conditions.

The organic aerosol is a mixture of different organic species. The vapor mass concentrations of these species in pure form span many orders of magnitude. Practically, these species can be modeled as a set of N-lumped volatile components, X_1, X_2, ..., X_N. The overall vapor mass concentration C* over the SOA film can be expressed as:

$$C^* = \sum_{i=1}^{n} x_i f_i C_i^0 \tag{S1}$$

The term x_i denotes the mole fraction of component i in the SOA film. In the simulation, different components are assumed to have the same effective molecular weight. In this case, x_i also denotes the mass fraction of component i. The term f_i is the activity coefficient. An ideal solution assumption is used herein (f_i = 1). The term C_i^0 is the vapor mass concentration or volatility of component i in pure form.

S1.1 Evaporation of SOA thin film in a gas-saturation flow cell

The mass loss rate for each volatility components in QCM cell can be described by:

$$\frac{dm_i}{dt} = -QMfx_iC_i^0;$$
$$m_{tot} = \sum_{i=1}^{N_1} m_i\,;\, m_i = m_{tot}x_i; \tag{S2}$$

where flow rate Q = 30 cm^3 min^{-1}, molecular weight M = 250 g mol^{-1}, activity coefficient f=1. m_i,x_i,C_i^0 represents the absolute mass, mole (mass) fraction, and volatility of component i. N_1 is the total volatility bin number chosen. In consideration of the sensitivity of the QCM and the particle concentration in PAM OFR, four volatility bins (≤10, 10^2, 10^3, 10^4 μg m^{-3}) were applied to

predict the mass decay behavior of isoprene and limonene SOA during dark aging. For the purpose of this study, species with a volatility of ≤10 μg m^{-3} were defined as less volatile organic species (LVOS), while those with a volatility of ≥100 μg m^{-3} were classified as more volatile organic species (MVOS). The initial x (x_0) was optimized and summarized in Table S7.

S1.2 Coupled photolytic aging and evaporation of SOA thin film in a gas-saturation flow cell

With UV light irradiation, a photolytic decay was applied to fresh species of each volatility bin, as described by:

$$\frac{dm_i}{dt} = -QMfx_iC_i^0 - j_i m_i \tag{S3}$$

with j_i representing the first-order photolysis rate for component i.

To represent the photolytic reactions, a transition matrix P was used to describe the products' mass distribution in different volatility bins from photolysis of different fresh species. The evaporation behavior of photolytic products was also considered. As a result, the mass change rate for photolytic products (aged species) was described by:

$$\frac{d\omega_j}{dt} = -QMfy_jC_j^1 + \sum_{i=1}^{N_1} p_{ij} m_i j_i$$
$$m_{tot} = \sum_{i=1}^{N_1} m_i + \sum_{j=1}^{N_2} \omega_j \,;\, m_i = m_{tot}x_i; \omega_i = m_{tot}y_i; y_{i,0} = 0 \tag{S4}$$

Where p_{ij} represents the element in a position [i,j] of transition matrix P. ω_j, y_j, C_j^1 represent the absolute mass, mole (mass) fraction in total film mass, and volatility of aged component j. N_2 is the number of volatility bins for aged species.

To further resolve the interplay between volatility and photolytic aging-related processes, three different model settings, sensitivity Model I, II, and III, have been examined.

In sensitivity Model I, the initial 4 volatility bins for fresh SOA were further split into eight bins at the same proportion, and two different photolysis rates were assigned to each portion, assuming the photolytic rates are orthometric to the volatility of fresh SOA species. The given initial states for sensitivity model I are described by:

$$x_0 = [\beta x_{10} \quad \beta x_{20} \quad \beta x_{30} \quad \beta x_{40} \quad (1-\beta)x_{10} \quad (1-\beta)x_{20} \quad (1-\beta)x_{30} \quad (1-\beta)x_{40}];$$
$$J = [j_1 \quad j_1 \quad j_1 \quad j_1 \quad j_2 \quad j_2 \quad j_2 \quad j_2]; \tag{S5}$$

Where β represents the mass fraction of fresh SOA that has a photolysis rate of j_1 while the other fraction1−β has a photolysis rate of j_2. Two different photolysis rates are chosen since they reflect the lowest degree of freedom that can effectively predict the measured mass profiles of SOA during photolytic aging.

The photolytic products in sensitivity Model I are assumed to be either LVOS ($C_0 \leq 10$ μg m^{-3})., which does not evaporate in a QCM flow cell, or gas, which escapes from particle films instantly ($C_0 \geq 10^6$ μg m^{-3}). A combination of aged species volatility bins and transition matrix P are shown as:

$$C^1 = [\leq 10 \quad \geq 10^6]\ \mu g\ m^{-3}$$
$$P_{model\ I} = \begin{bmatrix} p_1 & 1-p_1 \\ p_1 & 1-p_1 \\ p_1 & 1-p_1 \\ p_1 & 1-p_1 \\ p_1 & 1-p_1 \\ p_1 & 1-p_1 \\ p_1 & 1-p_1 \\ p_1 & 1-p_1 \end{bmatrix} \tag{S6}$$

Where p_1 represents the mass fraction of LVOS in all photolytic products. The optimized parameters β, j_1, j_2, p_1 for observed photolytic aging of laboratory SOA with 300 nm light are shown in Table S10. It's worth noting that the gas mass fraction was set as $1-p_1$ solely to filfull mass conservation. The choice of gas mass fraction, however, does not influence the model results for sensitivity Model I, II, and III because only particle or film mass was constrained here.

In sensitivity Model II, LVOS and MVOS in fresh SOA have different photolytic rates. The products are assumed to be either LVOS or gas. The settings for model II are shown as:

$$x_0 = [x_{10} \quad x_{20} \quad x_{30} \quad x_{40}]$$
$$J = [j_1 \quad j_2 \quad j_2 \quad j_2]$$
$$C^1 = [\leq 10 \quad \geq 10^6]\ \mu g\ m^{-3} \tag{S7}$$
$$P_{model\ II} = \begin{bmatrix} p_1 & 1-p_1 \\ p_1 & 1-p_1 \\ p_1 & 1-p_1 \\ p_1 & 1-p_1 \end{bmatrix}$$

Where j_1, j_2 represent the photolysis rates for fresh LVOS and fresh MVOS, respectively. p_1 represents the mass fraction of LVOS in all photolytic products. The optimized parameters j_1, j_2, p_1 for photolytic aging of laboratory SOA with 300 nm light are shown in Table S11.

In sensitivity Model III, LVOS and MVOS in fresh SOA have different photolytic rates as in model II. In addition, six volatility bins (≤10, 10^2, 10^3, 10^4, 10^5, ≥10^6 μg m^{-3}) were applied to the photolytic products. The settings for sensitivity model III are described by:

$$
\begin{gathered}
x_0 = [x_{10} \quad x_{20} \quad x_{30} \quad x_{40}] \\
J = [j_1 \quad j_2 \quad j_2 \quad j_2] \\
C^1 = [\leq 10 \quad 100 \quad 10^3 \quad 10^4 \quad 10^5 \quad \geq 10^6]\ \mu g\ m^{-3} \\
P_{\text{model III}} = \begin{bmatrix} p_1 & \theta p_2 & \theta p_2 & \theta p_2 & \theta p_2 & (1-4\theta)p_2 \\ 0 & \theta & \theta & \theta & \theta & 1-4\theta \\ 0 & \theta & \theta & \theta & \theta & 1-4\theta \\ 0 & \theta & \theta & \theta & \theta & 1-4\theta \end{bmatrix}; p_1 + p_2 = 1
\end{gathered} \quad \text{(S8)}
$$

Where j_1, j_2 represent the photolysis rates for fresh LVOS and fresh MVOS, respectively. p_1, p_2 represent the mass fraction of LVOS products and products with volatility ≥10^2 μg m^{-3} (MVOS + gas) from photolytic aging of fresh LVOS. It's assumed that no aged LVOS are formed through the photolysis of fresh MVOS. θ (<0.25) represents the mass fraction of one bin of aged MVOS among all aged products with volatility ≥10^2 μg m^{-3}. The same fraction was assumed for all 4 MVOS bins to reduce the degree of freedom of the model. Again, the mass of all aged LVOS and aged MVOS was explicitly modeled based on the evaporation behavior, the mass of the gas products, however, was assumed based on mass conservation and was a 'net' mass. The 'real' gas mass remained unknown. The optimized parameters j_1, j_2, p_1, θ for photolytic aging of laboratory SOA with 300 nm and 345 nm light are shown in Table S9.

Finally, only model sensitivity Model III can explain the measured SOA volatility transformation and is used as final model in the maintext.

S1.3 Coupled photolytic aging and evaporation of ambient OA in gas-saturation flow cell

Limited by the precision of measurements obtained for ambient particle aging experiments, the formation of MVOS for ambient OA samples could not be accurately determined. Here, we modeled the photolytic mass decay assuming 64% of the mass loss was through MVOS evaporation with Mode III, which is the averaged value derived for SOA LVOS photolytic aging.

Table S13 shows the modelled results for two types of SOA and ambient OA. It turned out that 15% of the ambient OA was rapidly photolyzed with complete mass loss while the remaining 85% photolyzed more slowly, and a relative mass fraction remaining of 60.3 ± 5.2% was determined. A relative mass fraction remaining of 60.3 ± 5.2% was higher than that determined for isoprene SOA LVOS (34.0 ± 19.1%) and limonene SOA LVOS (40.2 ± 4.7%).

S1.4 Coupled photolytic aging and evaporation of SOA in ambient atmosphere.

In the ambient atmosphere, the evaporation rate dm/dt (kg s^{-1}) of S/IVOCs from a spherical single particle of diameter D_p is described by:

$$\frac{dm}{dt} = 2\pi D_p D_A Fs(C_\infty - C_{eq});$$
$$F_S = \frac{0.75\alpha(1 + Kn)}{Kn^2 + Kn + 0.283\alpha Kn + 0.75\alpha}; \quad \text{(S9)}$$
$$K_n = \frac{2\lambda}{D_p}; \lambda = \frac{3D_A}{c_A};$$

Where D_p is the diameter of the particle. An initial diameter of 100 nm is used. D_A (= 8 × 10^{-6} m^2 s^{-1}) is the diffusivity of evaporated molecules in air. F_S is the Fuchs-Sutugin correction for noncontinuum effect and imperfect mass accommodation. α is the mass accommodation coefficient and equals unity here. Kn is the Knudsen number. λ is the molecule mean free path. c_A is the molecule mean velocity. C_∞ is the vapor mass concentration far away from the particle and C_{eq} is the equilibrium vapor mass concentration at the surface of the particle, which can be described as:

$$C_{eq} = xMf C^0 F_K;$$
$$F_K = \exp\left(\frac{4\sigma M}{RTD_p\rho}\right) \quad \text{(S10)}$$

F_K is the Kelvin effect correction factor. A surface tension value of σ = 0.072 N m^{-1} is used here. ρ is the material density of the particle. A density of 1400 kg m^{-3} is used.

By including the evaporation and photolytic aging processes together, the mass evolution of particles in the ambient atmosphere can be described by

$$\frac{dm_i}{dt} = 2\pi D_p D_A F_S(C_{i,\infty} - x_i Mf C_i^0 F_K) - j_i m_i \quad \text{(S11)}$$

$$\frac{d\omega_j}{dt} = 2\pi D_p D_A F_S \left(C_{j,\infty} - x_j M f C_j^0 F_K\right) + \sum_{i=1}^{N_1} p_{ij} m_i j_i$$

$$m_{tot} = \sum_{i=1}^{N_1} m_i + \sum_{j=1}^{N_2} \omega_j \,; m_i = m_{tot} x_i; \omega_i = m_{tot} y_i; y_{i,0} = 0$$

$$m_{tot} = \frac{\pi}{6} D_p^3$$

Where m_i is the mass of fresh species i among a total number of N_1, and ω_j is the mass of aged species j among a total number of N_2.

To start, we apply a volatility distribution of fresh SOA. These SOA species were allowed to partition and reach equilibrium at a specific particle concentration c_p, resulting in the formation of fresh SOA. The residence ratio of a species with volatility C_i^0 in the particle phase can be described by

$$\zeta_i = \frac{1}{1 + \frac{C_i^0}{c_p}}; \qquad \text{(S12)}$$

In this case, the decided volatility distribution at different initial SOA mass concentrations is the same because of the very low volatility applied. The fresh SOA was then subjected to solar irradiance within our thermodynamic model in ambient particles. It was assumed that p_1 of the fresh SOA materials would maintain their volatility, while $(1-p_1)4\theta$ of the mass would increase in volatility by two orders of magnitude. By covering the typical particle concentration level, the model settings for ambient OA photolytic aging in the ambient atmosphere are described:

$$C^0 = [10^{-8} \quad 10^{-6} \quad 10^{-4} \quad 10^{-2} \quad 1 \quad 100\,]\mu g\, m^{-3}$$

$$C^1 = [10^{-8} \quad 10^{-6} \quad 10^{-4} \quad 10^{-2} \quad 1 \quad 100\,]\mu g\, m^{-3}$$

$$J = [j_1 \quad j_1 \quad j_1 \quad 0 \quad 0 \quad 0]$$

$$P_{ambient} = \begin{bmatrix} p_1 & (1-p_1)2\theta & (1-p_1)2\theta & 0 & 0 & 0 \\ 0 & p_1 & (1-p_1)2\theta & (1-p_1)2\theta & 0 & 0 \\ 0 & 0 & p_1 & (1-p_1)2\theta & (1-p_1)2\theta & 0 \\ 0 & 0 & 0 & p_1 & (1-p_1)2\theta & 0 \\ 0 & 0 & 0 & 0 & 0 & 0 \end{bmatrix} \qquad \text{(S13)}$$

$C_i^0 \geq 100$ μg m^{-3} is assumed to be gas and is ignored in the model. For isoprene-derived SOA, here it was assumed that 22% (p_1) of the fresh SOA materials would maintain their volatility, while

52% ($4(1-p_1)$) of the mass would increase in volatility by 2 orders of magnitude. For the modelling results, refer to Figure S24.

S2 QCM film thickness and light attenuation ratio calculations

The average film thickness deposited on the QCM crystals in our experiments was estimated as $D=m/(\rho S)$, where m is the deposited mass, S is the sensor deposited area and ρ is the particle density. The light attenuation ratio through the film is described by $R_p = \exp(-MAC\times\rho\times D)$, where MAC is the mass absorption coefficient.

The deposited film mass, average film thickness, and light attenuation ratios are listed in Table S5. For limonene and isoprene SOA, absorption coefficients were taken from Romonosky et al.[56] For ambient particles, we adopted the upper limit of absorption coefficients reported across all investigated aerosols in Romonosky et al.[56] to represent a worst-case scenario due to data unavailability.

Table S1 Experimental conditions for SOA generation in PAM OFR. [a] Assume the average ambient [OH] = 1.5× 10^6 molecule cm^{-3} and [O_3] = 150 ppb.

SOA system	Precursor concentration (ppb)	Photochemical age (h)[a]	RH (%)/Temperature (˚C)
Isoprene + OH	1780	15	32/25
Limonene + O_3	125	3	31/22

Table S2 SMPS and HR-ToF-AMS measurement of SOA generated in PAM OFR. These values show the average value ± standard deviation.

SOA system	Mass conc	O:C	H:C	OSc	density/g cm^{-3}
Isoprene + OH	70	0.98±0.03	2.02±0.04	−0.06±0.05	1.40
Limonene + O_3	150	0.51±0.01	1.66±0.02	−0.64±0.02	1.25

Table S3 Information of ambient particle collection and HR-ToF-AMS measurement

Sample	Sampling time	collection time/h	Concentration/μg m^{-3}				O:C	H:C	OSc	Density/g cm^{-3}
			Total	Organics	Nitrate	Sulfate				
1	12th-14th Aug	49	10.2	8.43	0.22	1.16	0.67	1.49	−0.15	1.41
2	14th-17th Aug	69	11.0	8.79	0.28	1.42	0.69	1.48	−0.10	1.42
3	17th-19th Aug	49	10.4	8.40	0.34	1.21	0.68	1.48	−0.12	1.42
4	19th-21st Aug	48	7.0	5.35	0.21	1.06	0.64	1.52	−0.24	1.37
5	21st-23rd Aug	48	8.2	5.82	0.20	1.64	0.65	1.50	−0.20	1.39
6	23rd-25th Aug	45	6.8	5.16	0.18	1.06	0.58	1.54	−0.38	1.34
7	27th-29th Aug	42	9.6	8.14	0.23	0.91	0.59	1.53	−0.35	1.38
8	29th-31st Aug	44	6.9	5.53	0.26	0.78	0.59	1.53	−0.35	1.34

Table S4 OA dark aging and photolytic aging experiments conditions.

Aerosol type	Experimental type	Wavelength	Gas	RH/%	T/K	Mass/μg	Irradiance/W m^{-2}
Isoprene SOA	Dark aging S1	NA	Air	~60	294	21.8	NA
	Dark aging S2	NA	Air	<5	294	24.3	NA
	Photolytic aging S3	300 nm	Air	~60	294	22.9	47.1±1.2
	Photolytic aging S4	300 nm	Air	~60	294	19.3	41.1±1.2
	Photolytic aging S5	300 nm	Air	~60	294	22.1	27.3±1.4
	Photolytic aging S6	300 nm	Air	~60	294	14.9	34.8±1.6
	Photolytic aging S7	300 nm	N_2	~60	294	24.6	38.6±1.2
	Photolytic aging S8	300 nm	Air	<5	294	24.4	43.5±2.4
	Photolytic aging S9	300 nm	Air	<5	294	24.4	43.5±2.4
	Photolytic aging S10	300 nm	Air	<5	294	11.6	44.0±1.6
	Photolytic aging S11	345 nm	Air	~60	294	17.4	158.1±7.1
Limonene SOA	Dark aging S1	NA	Air	~80	294	17.4	NA
	Dark aging S2	NA	Air	~80	294	21.8	NA
	Dark aging S3	NA	Air	<5	294	17.4	NA
	Photolytic aging S4	300 nm	Air	~80	294	21.2	45.9±1.2
	Photolytic aging S5	300 nm	Air	~80	294	18.3	39.8±1.2
	Photolytic aging S6	300 nm	Air	~80	294	18.0	42.3±1.2
	Photolytic aging S7	300 nm	Air	~80	294	15.8	38.3±1.2
	Photolytic aging S8	300 nm	Air	~80	294	17.3	35.2±1.4
	Photolytic aging S9	300 nm	N_2	~80	294	19.0	36.2±1.2
	Photolytic aging S10	300 nm	Air	<5	294	21.2	59.2±2.4
	Photolytic aging S11	300 nm	Air	<5	294	17.0	57.8±1.4
	Photolytic aging S12	300 nm	Air	<5	294	27.9	46.3±1.7
	Photolytic aging S13	345 nm	Air	~80	294	18.1	202.9±9.6
	Photolytic aging S14	345 nm	Air	<5	294	19.1	207.4±9.7
Ambient	Photolytic aging S1	345 nm	Air	~60	294	8.2	140.1±24.2
	Photolytic aging S2	345 nm	Air	~60	294	10.9	207.7±12.1
	Photolytic aging S3	345 nm	Air	~60	294	3.8	216.1±9.6
	Photolytic aging S4	300 nm	Air	~60	294	2.9	44.7±2.4
	Photolytic aging S5	300 nm	Air	~60	294	7.0	49.5±2.4
	Photolytic aging S6	345 nm	Air	~60	294	7.5	189.6±9.6
	Photolytic aging S7	345 nm	Air	<5	294	5.5	155.8±7.2
	Photolytic aging S8	345 nm	Air	~60	294	3.6	13.3±0.0

Table S5 Sample masses, film thicknesses and light attenuation ratios (R_p) for OA dark aging and photolytic aging experiments.

Aerosol type	Experimental type	Mass/μg	Depth/nm	R_p
Isoprene SOA	Dark aging S1	21.8	198	0.99
	Dark aging S2	24.3	221	0.99
	Photolytic aging S3	22.9	208	0.99
	Photolytic aging S4	19.3	176	0.99
	Photolytic aging S5	22.1	201	0.99
	Photolytic aging S6	14.9	136	1.00
	Photolytic aging S7	24.6	224	0.99
	Photolytic aging S8	24.4	222	0.99
	Photolytic aging S9	24.4	222	0.99
	Photolytic aging S10	11.6	105	1.00
	Photolytic aging S11	17.4	158	0.99
Limonene SOA	Dark aging S1	17.4	177	0.99
	Dark aging S2	21.8	222	0.99
	Dark aging S3	17.4	177	0.99
	Photolytic aging S4	21.2	216	0.99
	Photolytic aging S5	18.3	186	0.99
	Photolytic aging S6	18.0	183	0.99
	Photolytic aging S7	15.8	161	0.99
	Photolytic aging S8	17.3	176	0.99
	Photolytic aging S9	19.0	194	0.99
	Photolytic aging S10	21.2	216	0.99
	Photolytic aging S11	17.0	173	0.99
	Photolytic aging S12	27.9	284	0.99
	Photolytic aging S13	18.1	184	0.99
	Photolytic aging S14	19.1	195	0.99
Ambient	Photolytic aging S1	8.2	74	0.77
	Photolytic aging S2	10.9	98	0.71
	Photolytic aging S3	3.8	34	0.89
	Photolytic aging S4	2.9	27	0.93
	Photolytic aging S5	7.0	64	0.84
	Photolytic aging S6	7.5	71	0.79
	Photolytic aging S7	5.5	51	0.84
	Photolytic aging S8	3.6	34	0.89

Table S6 Chemical composition determined by nano-DESI-HRMS measurements and κ values determined by QCM measurements for isoprene SOA and limonene SOA photolytic aging under 300 nm light

	Isoprene SOA			Limonene SOA		
	Fresh	Aged (dry)	Aged (humid)	Fresh	Aged (dry)	Aged (humid)
Molecular weight	252	246	259	267	338	266
Carbon number	9.3	9.7	9.7	12.2	14.7	11.2
Oligomer-to-monomer ratio	2.03	4.09	2.90	0.51	1.61	0.65
O:C	0.90	0.77	0.88	0.53	0.64	0.69
H:C	1.62	1.57	1.52	1.51	1.40	1.40
OSc	0.19	−0.03	0.25	−0.44	−0.12	−0.03
κ	0.18±0.02	0.14±0.01	0.165±0.02	0.056±0.003	0.06±0.003	0.11±0.01

Table S7 Optimized initial mole/mass fraction (x_0) for fresh SOA generated in laboratory under different humidity. [a] The S/IVOCs mass yield for different volatility bins from Zhang et al.[71] are listed for comparison. The values show volatility distribution of fresh SOA in equilibrium under a particle mass of 150 μg m^{-3} .

SOA type	RH/%	Volatility(C^0)/μg m^{-3}			
		≤10	100	1000	10000
Isoprene SOA	~60<br><5	46.0%	26.7%	26.0%	1.4%
Limonene SOA	~80	54.7%	27.0%	17.6%	0.8%
	<5	75.4%	21.0%	3.4%	0.2%
Limonene SOA[a]	<10	70.0%	21.0%	9.0%	0.0%

Table S8 The measured mass change and normalized mass loss rate (NMLR) change for laboratory SOA photolytic aging. Subscript 0 and 1 refer to before and after evaporation respectively. The prediction is made by model III with the same parameterization determined as in Table S9.

SOA type	M_0/μg	M_1/μg	Evaporation fraction loss	Measured/10^{-4} s^{-1} NMLR$_0$	NMLR$_1$	Predicted NMLR$_1$/10^{-4} s^{-1}
Isoprene SOA	25.36	15.35±0.09	0.37±0.00	1.60±0.00	0.48±0.04	0.60
Limonene SOA	15.79	13.16±0.08	0.17±0.01	0.84±0.01	0.52±0.07	0.58

Table S9 The optimized parameters for sensitivity model III (final model) for photolytic aging of laboratory SOA with UV lights at wavelenths of 300 nm and 345 nm.

SOA type	Aging wavelength	RH	Sensitivity Model III			
			J_1/s^{-1}	J_2/s^{-1}	P_1	θ
Isoprene SOA	300 nm	<5	4.1×10^{-4}	4.62×10^{-5}	0.70	0.16
	300 nm	~60	5.06×10^{-4}	3.62×10^{-5}	0.17	0.11
	345 nm	~60	2.28×10^{-4}	2.72×10^{-5}	0.28	0.15
Limonene SOA	300 nm	<5	6.28×10^{-4}	1.41×10^{-4}	0.52	0.16
	300 nm	~80	5.02×10^{-4}	8.45×10^{-5}	0.33	0.17
	345 nm	~80	5.02×10^{-5}	8.45×10^{-5}	0.40	0.23

Table S10 The optimized parameters for sensitivity model I for photolytic aging of laboratory SOA with 300 nm light.

SOA type	UV wavelength	RH	Sensitivity Model I			
			β	J_1/s^{-1}	J_2/s^{-1}	P_1
Isoprene SOA	300 nm	~60	0.50	3.38×10^{-4}	1.51×10^{-5}	0.08
		<5	0.54	4.14×10^{-4}	2.31×10^{-5}	0.40
Limonene SOA		~80	0.55	3.14×10^{-4}	3.52×10^{-5}	0.24
		<5	0.53	2.66×10^{-4}	5.30×10^{-5}	0.45

Table S11 The optimized parameters for sensitivity model II for photolytic aging of laboratory SOA with 300 nm light.

SOA type	UV wavelength	RH	Sensitivity Model II		
			J_1/s^{-1}	J_2/s^{-1}	P_1
Isoprene SOA	300 nm	~60	3.38×10^{-4}	2.72×10^{-5}	0.08
		<5	4.14×10^{-4}	4.62×10^{-5}	0.40
Limonene SOA		~80	3.14×10^{-5}	3.52×10^{-5}	0.24
		<5	3.04×10^{-4}	1.06×10^{-4}	0.45

Table S12 Comparison of σ_{Φ} calculated for different composition of different OA with OA absorption cross section. The SOA absorption cross sections are taken from Romonosky et al.[56] assuming molecular weight = 250 g cm^{-3}.

OA type	Composition	$\sigma_{\Phi 300\ nm}$/photon^{-1} cm^2	$\sigma_{\Phi 345\ nm}$/ photon^{-1} cm^2	$\sigma_{300\ nm}$/cm^2	$\sigma_{345\ nm}$/cm^2
Isoprene SOA	MVOS	$7.3(\pm1.2) \times 10^{-20}$	8.2×10^{-21}	9.8×10^{-20}	7.7×10^{-21}
	LVOS	$6.2(\pm1.0) \times 10^{-21}$	9.8×10^{-22}		
Limonene SOA	MVOS	$7.0(\pm0.3) \times 10^{-20}$	1.4×10^{-21}	1.3×10^{-19}	9.1×10^{-21}
	LVOS	$1.3(\pm0.1) \times 10^{-20}$	2.4×10^{-22}		
Ambient OA	LVOS 1	$4.2(\pm1.4) \times 10^{-20}$	$9.3(\pm2.4) \times 10^{-21}$	NA	
	LVOS 2	$5.9(\pm0.4) \times 10^{-21}$	$1.5(\pm0.3) \times 10^{-21}$		

Table S13 Determined photolysis rates under standard solar radiation for different composition of different OA under humid conditions. Referenced j_{NO2} = 3.0 × 10^{-3} s^{-1} under standard solar radiation.

OA type	Composition	Initial mass fraction	j_{amb}/s^{-1}	j_{amb} : j_{NO2}/%	τ_{amb}/h	MVOS yield	LVOS yield
Isoprene	MVOS	0.54	3.0±0.5 × 10^{-5}	1.00±0.17	9.3±1.5	0.44	0
SOA	LVOS	0.46	3.5±0.6 × 10^{-6}	0.12±0.02	79±14	0.29±0.08	0.34±0.19
Limonene	MVOS	0.45	7.8±0.4 × 10^{-6}	0.26±0.01	36±2	0.68	0
SOA	LVOS	0.55	1.3±0.1 × 10^{-6}	0.04±0.01	214±16	0.41±0.03	0.40±0.05
Ambient	LVOS 1	0.15	3.4±1.4 × 10^{-5}	1.13±0.47	8.2±3.4	0.64	0
OA	LVOS 2	0.85	5.4±1.2 × 10^{-6}	0.18±0.04	51±11	0.26±0.03	0.6±0.05

List of supplementary figures

Figure S1. Schematic diagram of QCM sample collection.

Figure S2. Schematic diagram of the apparatus for aging experiments in a QCM flow cell.

Figure S3. Absolute and baseline-corrected frequency for different overtones during a typical photolytic aging experiment under dry conditions. The shaded area represents the period when the QCM sensor is loaded with a particle film.

Figure S4. Comparison of spectra between laboratory lamps and standard solar radiation. The irradiance shown for 300 nm lamp is 40 W m^{-2} and for 345 nm lamp is 150 W m^{-2}, both were typical values used for photolytic aging experiments.

Figure S5. Relative mass loss rate of isoprene SOA as a function of flow rate in a QCM flow cell, normalized by the mass loss rate at 30 sccm flow rate.

Figure S6. Baseline-corrected frequency profiles for aging experiments under different RH. Panel A shows dark aging under dry conditions. Panel B shows dark aging under humid conditions. Blue shading indicates time periods with elevated RH, where the particle film absorbs water and frequency decreases. Panel C presents photolytic aging under dry conditions, with t=0 representing the time the lamp was turned on for the first time. Pink shading represents the light-on periods. Panel D illustrates photolytic aging under humid conditions, with purple shading indicating time periods with both elevated RH and UV irradiation.

Figure S7. Temperature disturbance caused by UV irradiation in the QCM sensor. Panel A shows the change in saturation vapor pressure, $e_s(T+dT)/e_s(T)$, at the sensor surface when an irradiance is applied. The RH of the air remains constant, while the RH at the sensor surface decreases due to increased temperature. The RH change (RH(T+ dT)/RH(T)) is derived by assuming a constant hygroscopicity within a short period and observing the changes in absorbed water mass. By definition, RH(T)/RH(T+ dT) = $e_s(T+ dT)/e_s(T)$. Since the changes in absorbed water mass due to temperature disturbance are small and influenced by the instability of the humidified flow, multiple trials are required to obtain an average value. Considering the controlled temperature of the QCM (294±0.01 K), the function es(T+ dT)/es(T) as a function of dT within

294–295 K is approximated by $e_s(T+ dT)/e_s(T) = 0.052$ dT/K. Panel B shows the correlation between the temperature response of the QCM sensor and the irradiated light (represented as the direct frequency response). An equation dT/K = 0.065 dF/Hz is used to correct the RH when calculating κ during photolytic aging.

Figure S8. Mass of adsorbed water onto the QCM sensor as a function of RH.

Figure S9. Correlation between the direct frequency response and the measured UV irradiation obtained by a photometer. The linearity of this response was confirmed up to 300 W m^{-2}. The derived slope of 12.075 W m^{-2} Hz^{-1} can be extrapolated and used to calculate the irradiance during each aging experiment.

Figure S10. Mass loss rate of fresh limonene ozonolysis SOA in the QCM flow cell as a function of RH.

Figure S11 Temporal decay of mass fraction for isoprene photooxidation SOA (A) and limonene ozonolysis SOA (B) normalized to the same photon flux. For humid conditions, the isoprene SOA experiments were conducted at approximately 60% RH, while the limonene SOA experiments were at around 80% RH. An uncertainty of 0.75 µg was used for a single measurement, as shown by the shaded area.

Figure S12. Temporal decay of mass fraction for isoprene photooxidation SOA (A) and limonene ozonolysis SOA (B) during 300 nm photolytic aging under humid conditions but in different gas in a QCM flow cell. For humid conditions, the isoprene SOA experiments were conducted at approximately 60% RH, while the limonene SOA experiments were at around 80% RH. An uncertainty of 0.75 µg was used for a single measurement, while for multiple measurements, a collective uncertainty was applied, as shown by the shaded area. The time resolution for each line is 30 min.

Figure S13. Mass loss rate elevation for isoprenen SOA under humid condition by replacing N_2 with zero air after long-term photolytic aging.

Figure S14. The measured and modeled intrinsic volatility of isoprene SOA under dry conditions (A) and humid conditions (B) and limonene under dry conditions (C) and humid

conditions (D). The shown intrinsic volatility was for the last 150 s of each light-off period. The intrinsic volatility was modeled using a volatility box model incorporating photolytic volatility transformation with three different settings. Details of the three sensitivity models are provided in Text S1.

Figure S15. Comparison of hygroscopicity (κ) profiles as a function of mass fraction remaining for isoprene SOA (A) and limonene SOA (B) across different replications. For limonene SOA, sample S6 started with RH ~ 60% for the first 3 h, resulting in higher κ. The values are binned averages at 2% mass fraction intervals.

Figure S16. Comparison of hygroscopicity (κ) before and after aging as a function of RH for isoprene SOA (A) and limonene SOA (B). The κ values after 300 nm photolytic aging of isoprene SOA are taken from the trial with approximately ~30% mass fraction remaining.

Figure S17. Oxygen-to-carbon (O:C) ratio vs carbon number distribution for fresh and photolytic aged isoprene SOA. The volatility is calculated using carbon number and O:C.

Figure S18. The same as Figure S17 but for limonene SOA

Figure S19. Temporal decay of ambient OA mass fraction remaining during photolytic aging under humid conditions. High light intensity trials are linearly scaled to low light intensity.

Figure S20. Hygroscopicity (κ) profiles as a function of mass fraction remaining for different ambient particle samples. The values are binned averages at 2% mass fraction intervals.

Figure S21. Comparison of measured and modeled evaporation behavior for two types of SOA. Panel A and B show the mass loss rate as a function of the mass fraction remaining for isoprene SOA (A) and limonene SOA (B) under different RH. Panels C and D show the corresponding mass decay temporal profiles for limonene SOA (C) and isoprene SOA (D) under humid conditions.

Figure S22. The same as Figure 2b, c–f but for photolytic aging of isoprene SOA (A and C) and limonene SOA (B and D) at 345 nm light under humid conditions.

Figure S23. The product of absorption cross-section and quantum yield σ_Φ (unit: photons^{-1} cm^2) values for limonene SOA, isoprene SOA, and ambient OA under humid conditions as a function of wavelength. The absorption cross sections for limonene SOA and isoprene SOA were also shown for comparison.

Figure S24. Photolytic aging model results of ambient isoprene-derived SOA under solar irradiance. A. Modeled temporal mass fraction decay for isoprene SOA under solar irradiance with different initial particle masses. B. Modeled Volatility Distribution for isoprene SOA before and after 240 h of photolytic aging under solar radiation normalized by initial particle concentration.

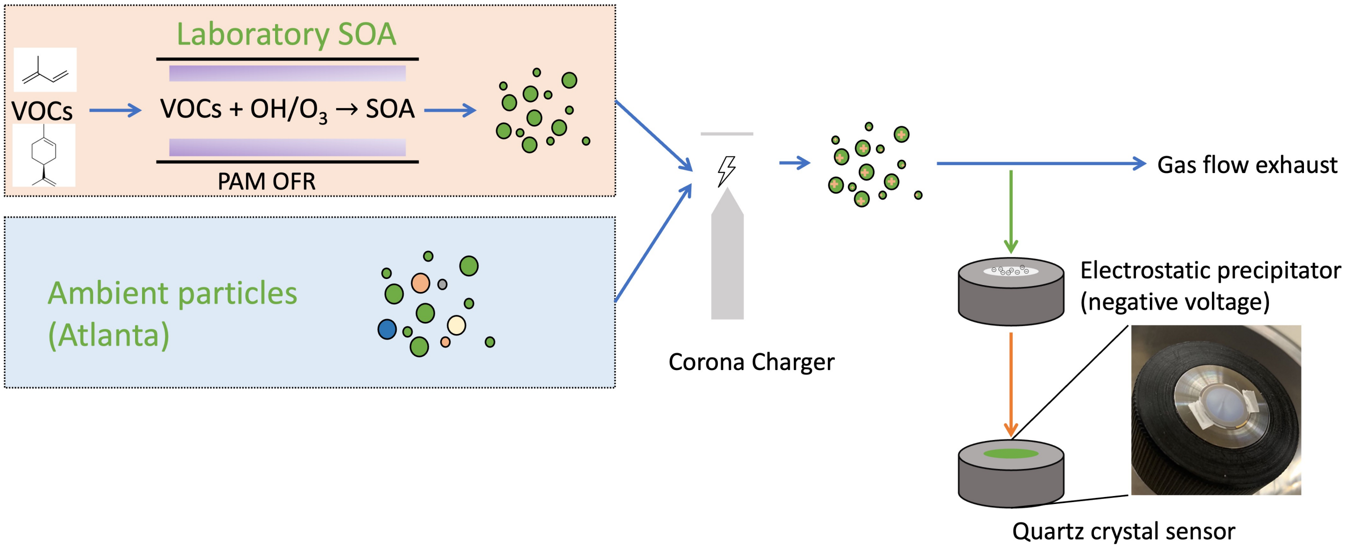
Laboratory SOA
VOCs
VOCs + OH/O$_3$ → SOA
PAM OFR
Ambient particles
(Atlanta)
Corona Charger
Gas flow exhaust
Electrostatic precipitator
(negative voltage)
Quartz crystal sensor

Figure S1

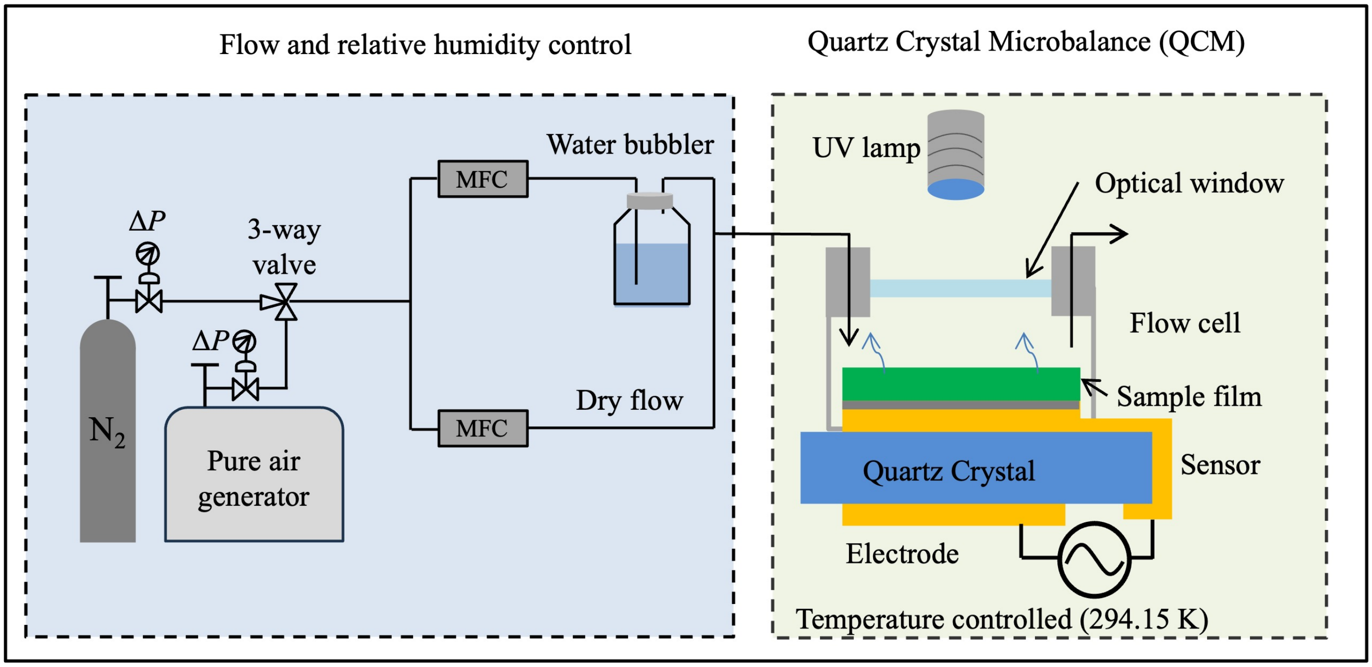
Flow and relative humidity control
Quartz Crystal Microbalance (QCM)
Water bubbler
MFC
ΔP
3-way
valve
ΔP
N_2
Pure air
generator
Dry flow
MFC
UV lamp
Optical window
Flow cell
Sample film
Quartz Crystal
Sensor
Electrode
Temperature controlled (294.15 K)

Figure S2

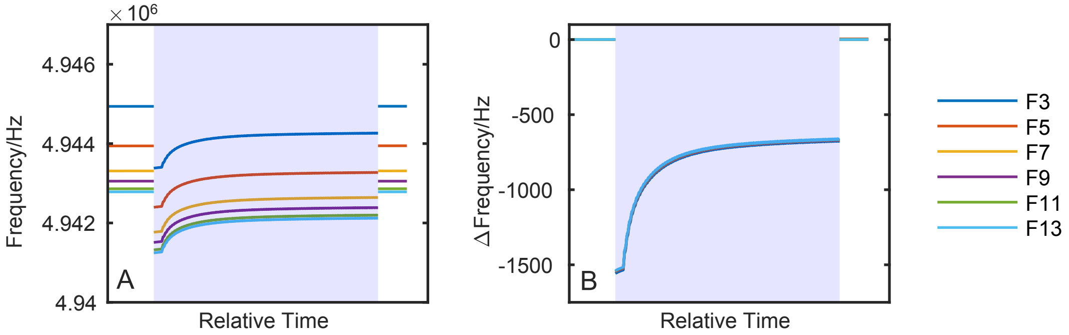
×10^6
4.946
4.944
4.942
4.94
Frequency/Hz
A
Relative Time
0
-500
-1000
-1500
ΔFrequency/Hz
B
Relative Time
F3
F5
F7
F9
F11
F13

Figure S3

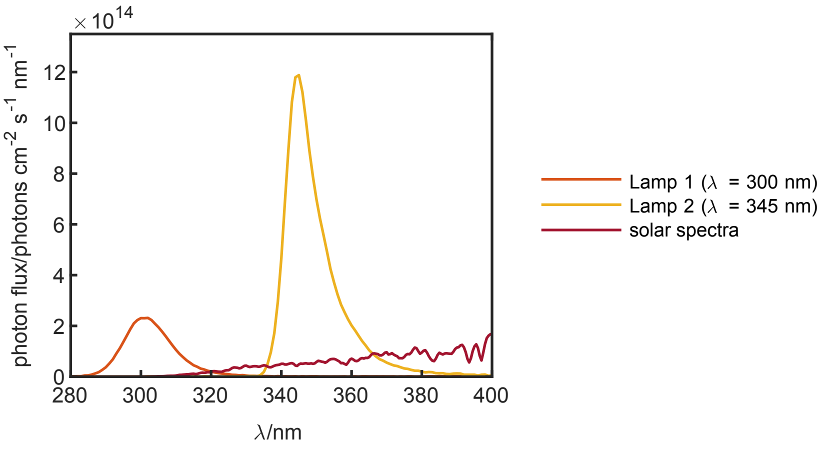
× 10^14
photon flux/photons cm^-2 s^-1 nm^-1
12
10
8
6
4
2
0
280
300
320
340
360
380
400
λ/nm
Lamp 1 (λ = 300 nm)
Lamp 2 (λ = 345 nm)
solar spectra

Figure S4

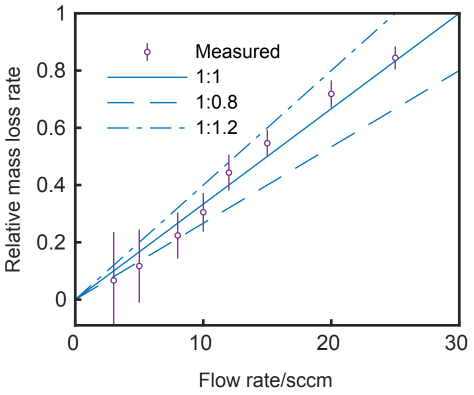

Relative mass loss rate
Flow rate/sccm
Measured
1:1
1:0.8
1:1.2
0
0.2
0.4
0.6
0.8
1
0
10
20
30

Figure S5

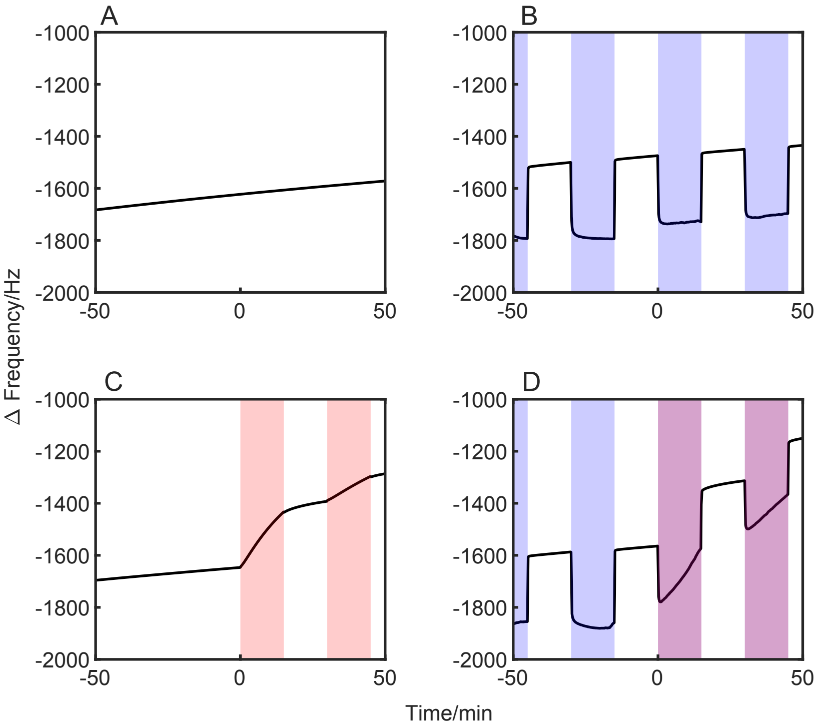
A
B
C
D
-1000
-1200
-1400
-1600
-1800
-2000
-50
0
50
Δ Frequency/Hz
Time/min

Figure S6

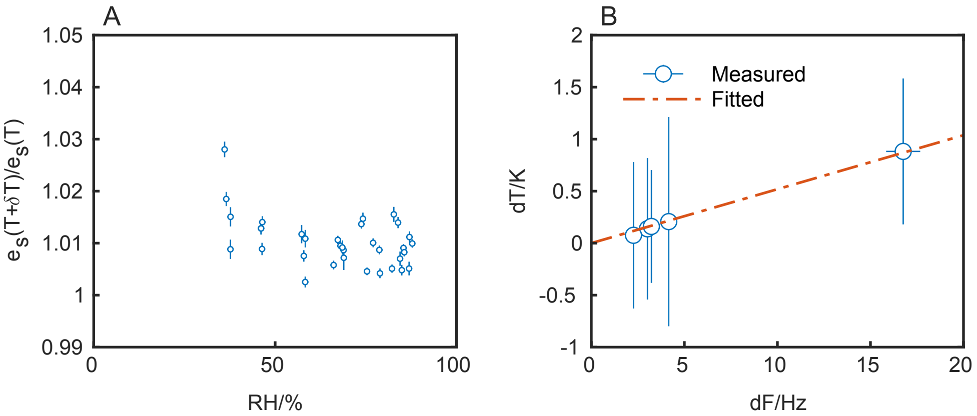

A
1.05
1.04
1.03
1.02
1.01
1
0.99
0
50
100
RH/%
$e_s(T+\delta T)/e_s(T)$
B
2
1.5
1
0.5
0
-0.5
-1
0
5
10
15
20
dF/Hz
dT/K
Measured
Fitted

Figure S7

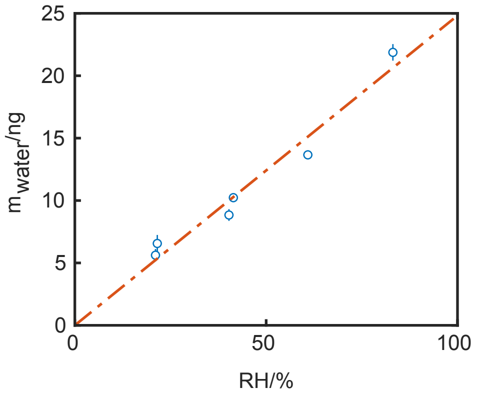

Figure S8

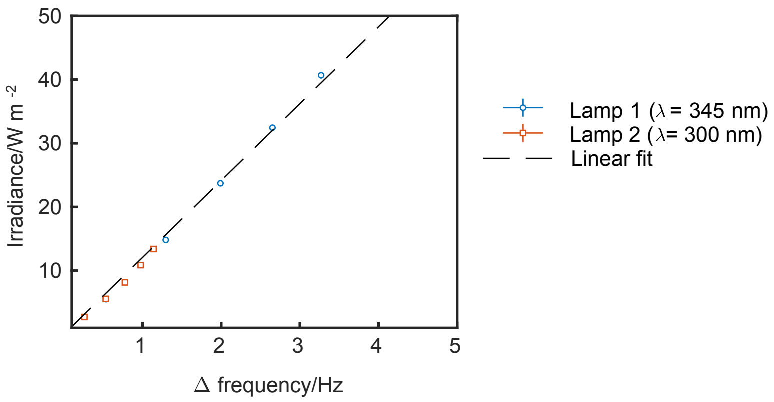
Irradiance/W m -2
50
40
30
20
10
1
2
3
4
5
Δ frequency/Hz
Lamp 1 (λ = 345 nm)
Lamp 2 (λ= 300 nm)
Linear fit

Figure S9

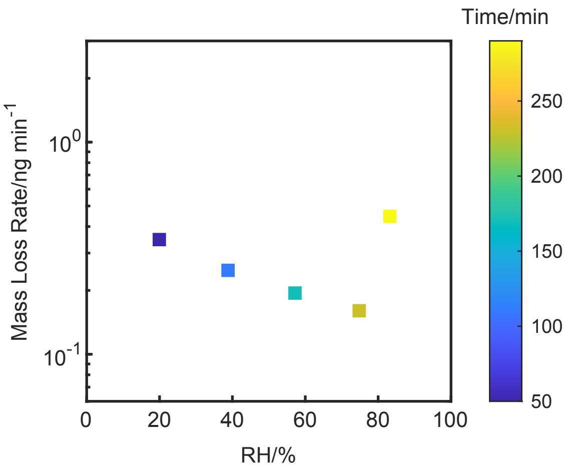
Time/min
Mass Loss Rate/ng min^{-1}
10^0
10^{-1}
0
20
40
60
80
100
RH/%
250
200
150
100
50

Figure S10

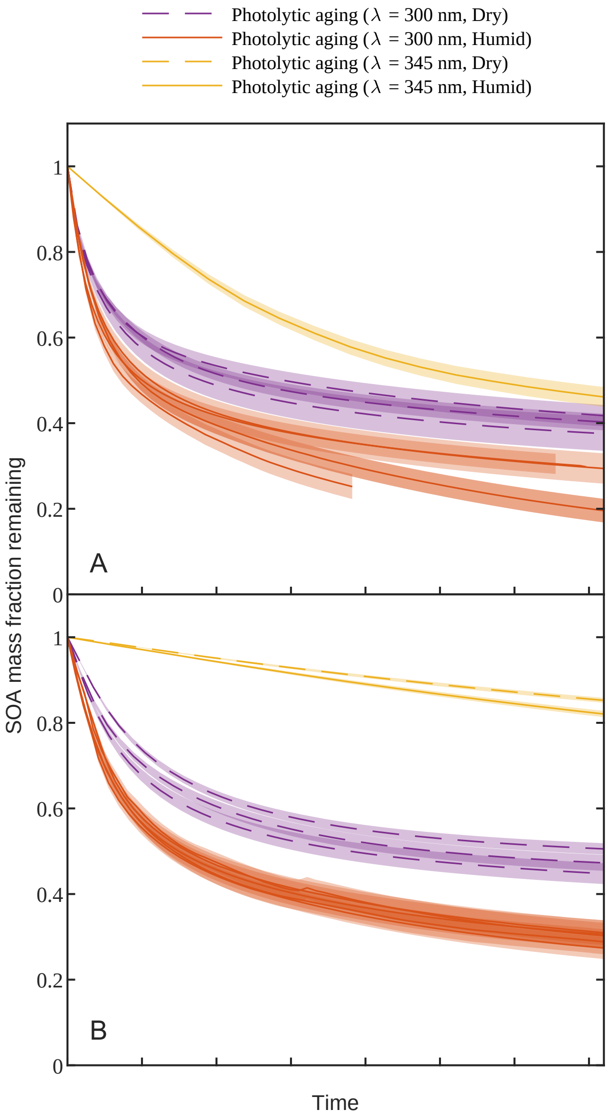

Photolytic aging (λ = 300 nm, Dry)
Photolytic aging (λ = 300 nm, Humid)
Photolytic aging (λ = 345 nm, Dry)
Photolytic aging (λ = 345 nm, Humid)
SOA mass fraction remaining
Time
A
B
0
0.2
0.4
0.6
0.8
1

Figure S11

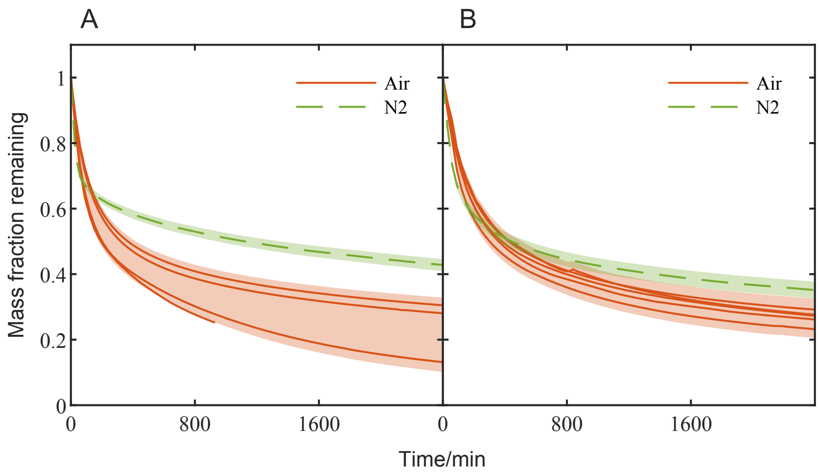
A
B
Air
N2
Air
N2
Mass fraction remaining
0
0.2
0.4
0.6
0.8
1
0
800
1600
0
800
1600
Time/min

Figure S12

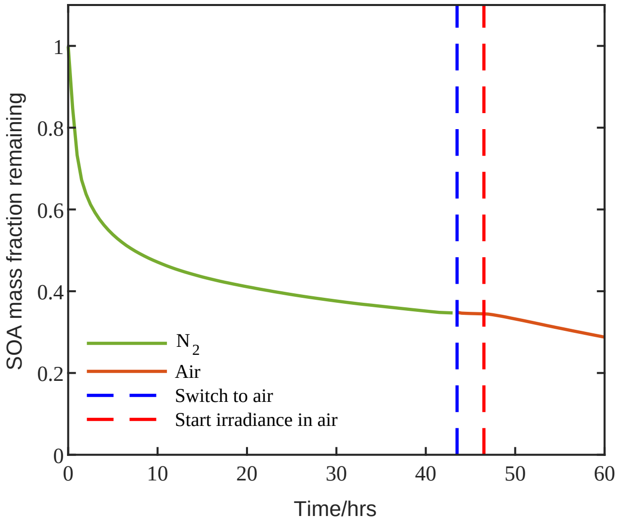

SOA mass fraction remaining
1
0.8
0.6
0.4
0.2
0
0
10
20
30
40
50
60
Time/hrs
N 2
Air
Switch to air
Start irradiance in air

Figure S13

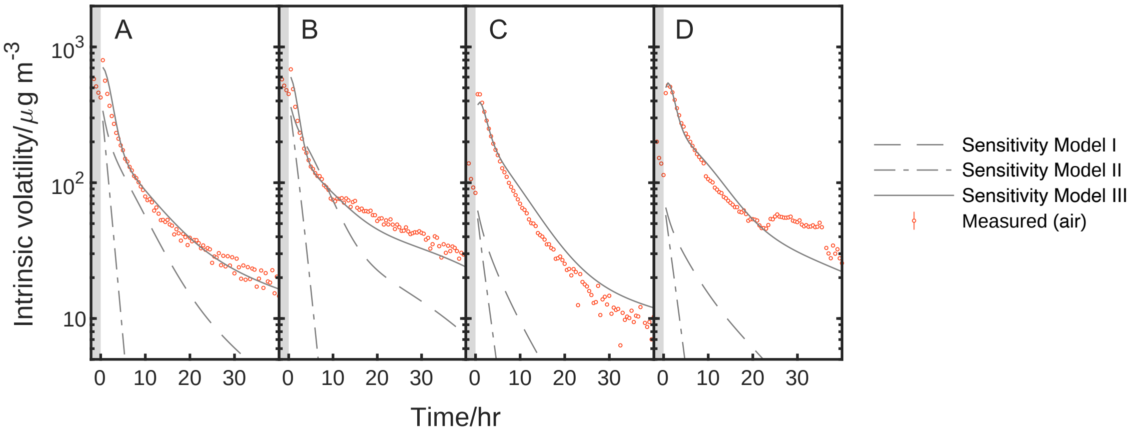
A
B
C
D
Intrinsic volatility/μg m^{-3}
10^3
10^2
10
0
10
20
30
Time/hr
Sensitivity Model I
Sensitivity Model II
Sensitivity Model III
Measured (air)

Figure S14

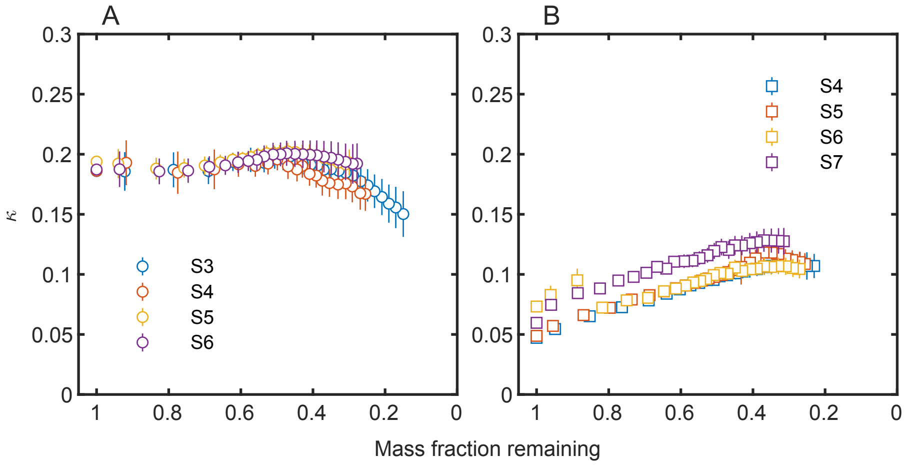

A
B
0.3
0.25
0.2
0.15
0.1
0.05
0
κ
1
0.8
0.6
0.4
0.2
0
S3
S4
S5
S6
S7
Mass fraction remaining

Figure S15

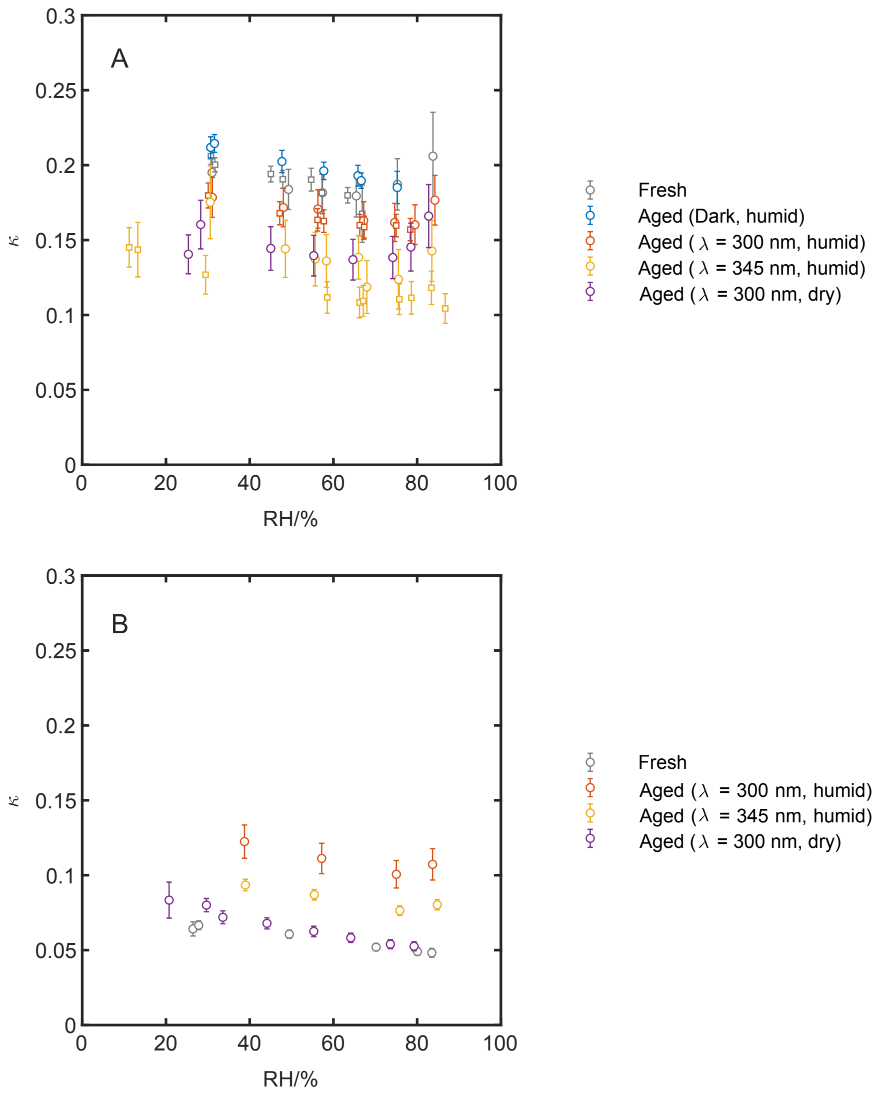
A
B
0.3
0.25
0.2
0.15
0.1
0.05
0
0
20
40
60
80
100
RH/%
κ
Fresh
Aged (Dark, humid)
Aged (λ = 300 nm, humid)
Aged (λ = 345 nm, humid)
Aged (λ = 300 nm, dry)

Figure S16

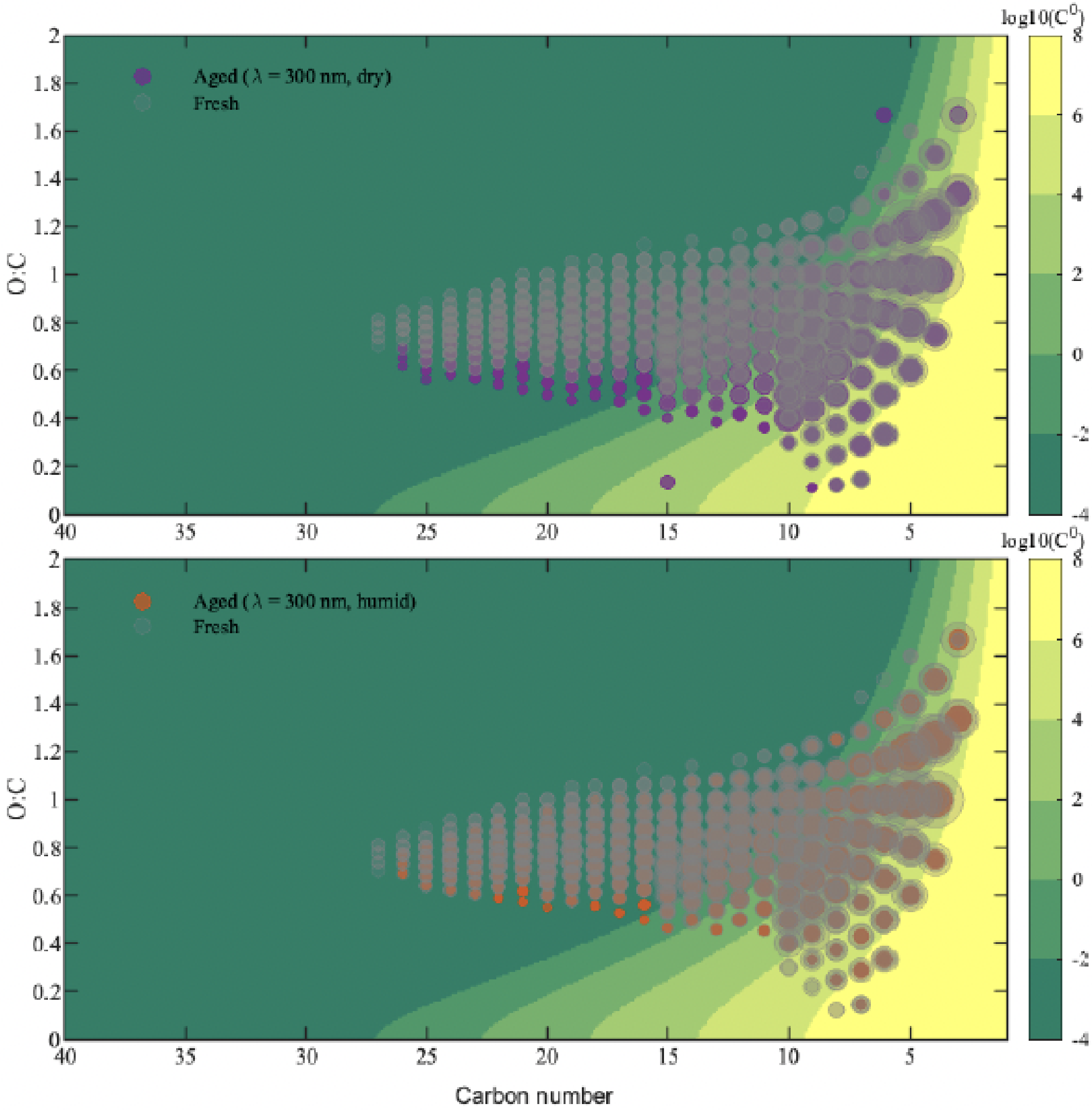

log10(C⁰)
Aged (λ = 300 nm, dry)
Fresh
O:C
Aged (λ = 300 nm, humid)
Fresh
O:C
Carbon number

Figure S17

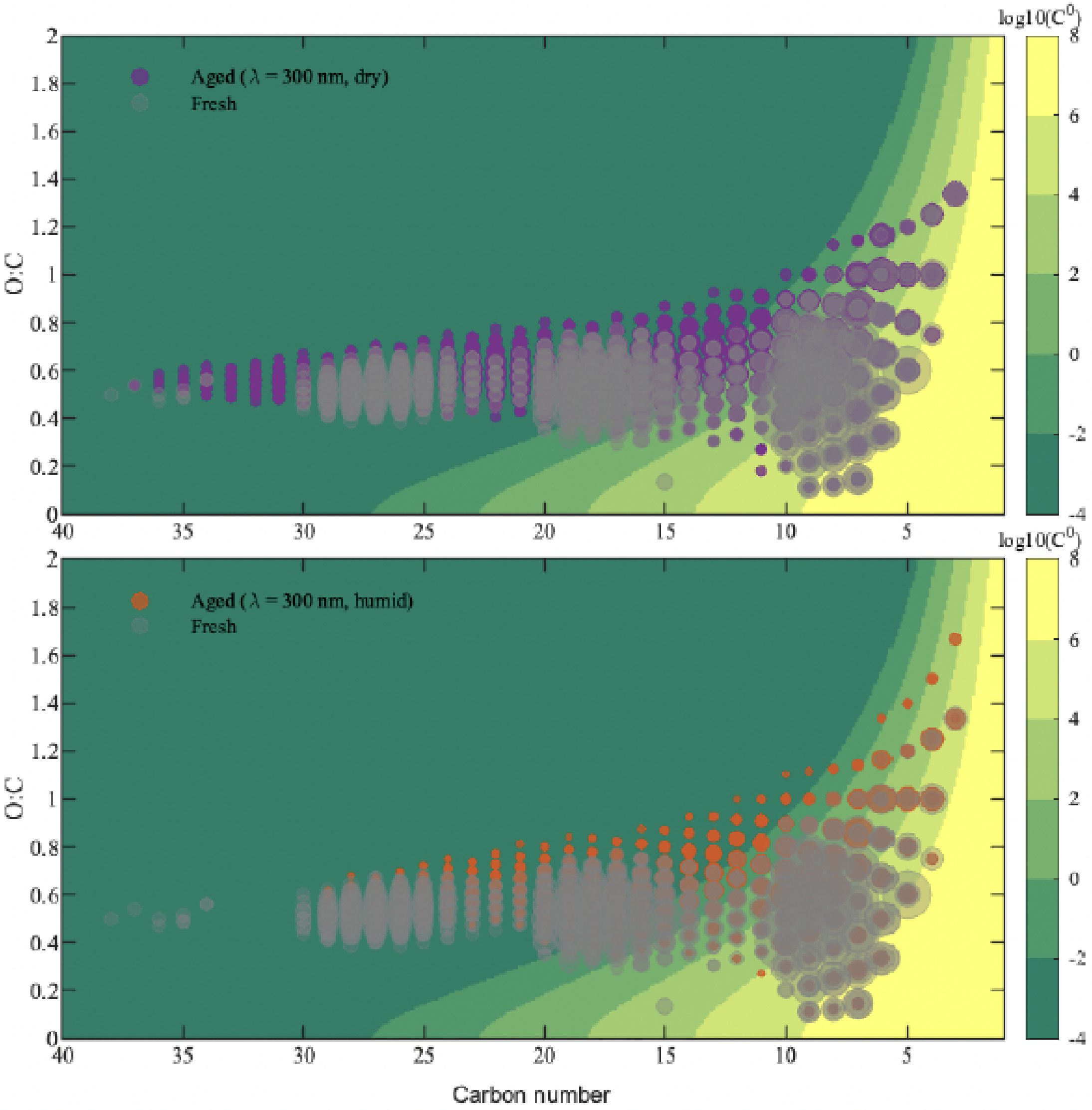

log10(C0)
Aged (λ = 300 nm, dry)
Fresh
O:C
Aged (λ = 300 nm, humid)
Fresh
O:C
Carbon number

Figure S18

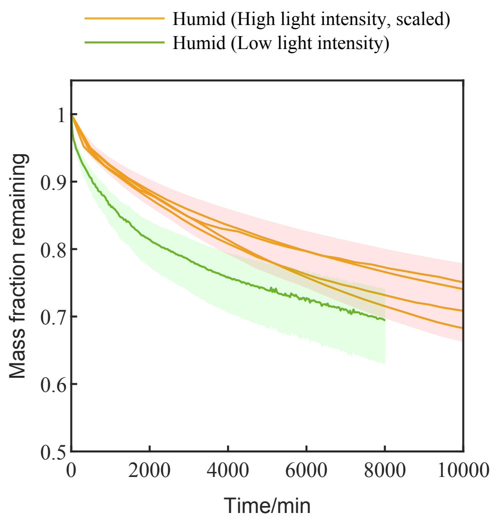

Humid (High light intensity, scaled)
Humid (Low light intensity)
Mass fraction remaining
1
0.9
0.8
0.7
0.6
0.5
0
2000
4000
6000
8000
10000
Time/min

Figure S19

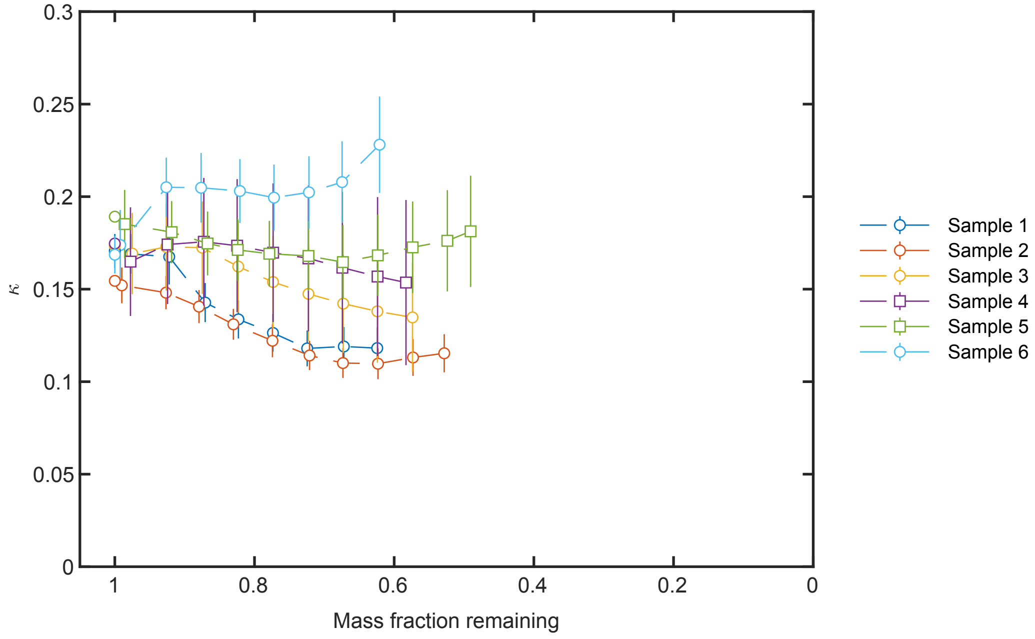
0.3
0.25
0.2
0.15
0.1
0.05
0
κ
1
0.8
0.6
0.4
0.2
0
Mass fraction remaining
Sample 1
Sample 2
Sample 3
Sample 4
Sample 5
Sample 6

Figure S20

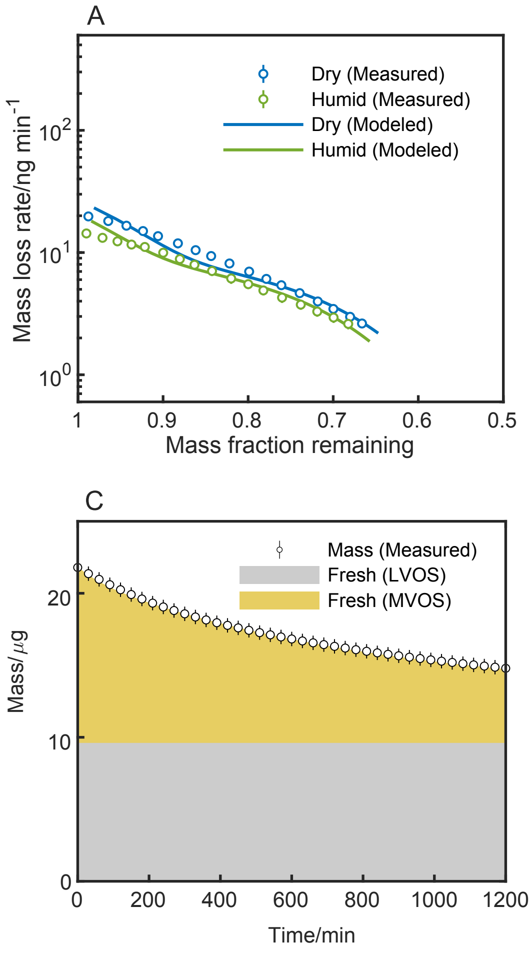

A
Dry (Measured)
Humid (Measured)
Dry (Modeled)
Humid (Modeled)
Mass loss rate/ng min^{-1}
10^2
10^1
10^0
1
0.9
0.8
0.7
0.6
0.5
Mass fraction remaining
B
Dry (Measured)
Humid (Run 1)
Humid (Run2)
Dry (Modeled)
Humid (Modeled)
Mass loss rate/ng min^{-1}
10^1
10^0
10^{-1}
1
0.9
0.8
0.7
0.6
0.5
Mass fraction remaining
C
Mass (Measured)
Fresh (LVOS)
Fresh (MVOS)
Mass/μg
20
10
0
0
200
400
600
800
1000
1200
Time/min
D
Mass (Measured)
Fresh (LVOS)
Fresh (MVOS)
Mass/μg
20
10
0
500
1000
1500
2000
Time/min

Figure S21

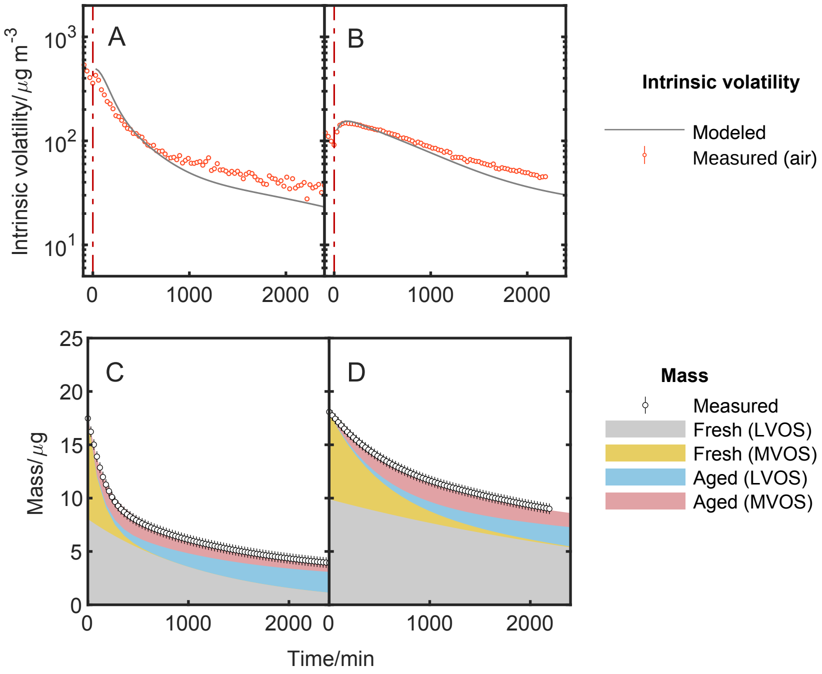

A
B
C
D
Intrinsic volatility/μg m-3
10^3
10^2
10^1
0
1000
2000
Intrinsic volatility
Modeled
Measured (air)
Mass/μg
25
20
15
10
5
Time/min
Mass
Measured
Fresh (LVOS)
Fresh (MVOS)
Aged (LVOS)
Aged (MVOS)

Figure S22

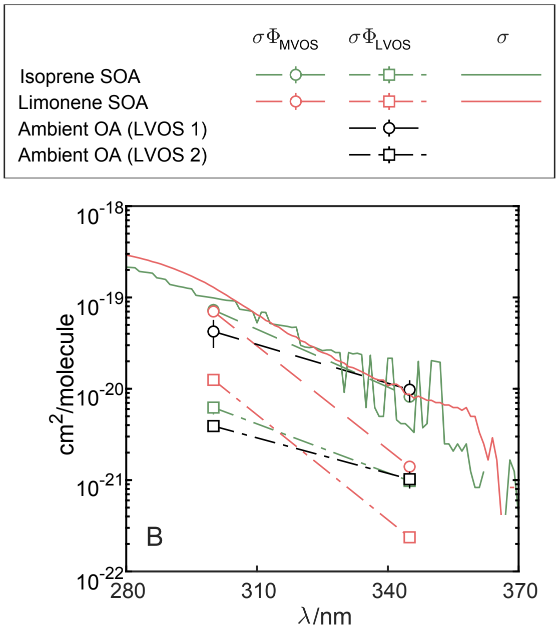

σΦMVOS
σΦLVOS
σ
Isoprene SOA
Limonene SOA
Ambient OA (LVOS 1)
Ambient OA (LVOS 2)
cm2/molecule
10-18
10-19
10-20
10-21
10-22
280
310
340
370
λ/nm
B

Figure S23

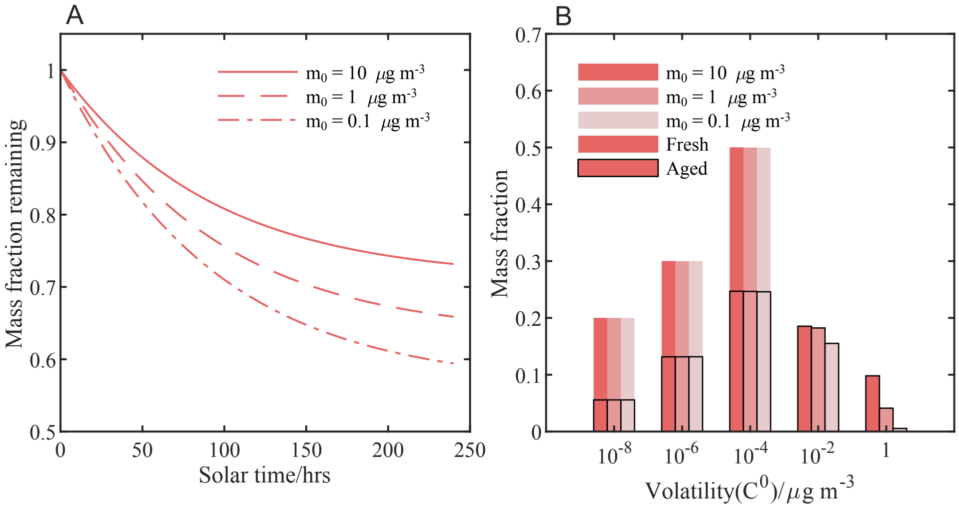
A
B
m0 = 10 μg m-3
m0 = 1 μg m-3
m0 = 0.1 μg m-3
Mass fraction remaining
Solar time/hrs
Fresh
Aged
Mass fraction
Volatility(C0)/μg m-3

Figure S24
